# Paired guide RNA CRISPR-Cas9 screening for protein-coding genes and lncRNAs involved in transdifferentiation of human B-cells to macrophages

**DOI:** 10.1186/s12864-022-08612-7

**Published:** 2022-05-26

**Authors:** Carme Arnan, Sebastian Ullrich, Carlos Pulido-Quetglas, Ramil Nurtdinov, Alexandre Esteban, Joan Blanco-Fernandez, Estel Aparicio-Prat, Rory Johnson, Sílvia Pérez-Lluch, Roderic Guigó

**Affiliations:** 1 Centre for Genomic Regulation (CRG), The Barcelona Institute for Science and Technology, Barcelona (BIST), Dr. Aiguader 88, 08003 Barcelona, Catalonia Spain; 2 Department of Medical Oncology, Bern University Hospital, University of Bern, Inselspital, Switzerland; 3 Department for BioMedical Research, University of Bern, Bern, Switzerland; 4 Present address: Department of Research and Innovation, “la Caixa” Foundation, Barcelona, Catalonia Spain; 5 Present address: Department of Immunobiology, University of Lausanne, Epalinges, Switzerland; 6 School of Biology and Environmental Science, University College Dublin, Dublin, Ireland; 7 Conway Institute for Biomolecular and Biomedical Research, University College Dublin, Dublin, Ireland; 8 Universitat Pompeu Fabra (UPF), Barcelona, Catalonia Spain

**Keywords:** CRISPR-Cas9 screening, Cellular transdifferentiation, Paired guide RNA (pgRNA), Long non-coding RNA (lncRNA), Genome editing, Human B-cells, Macrophages

## Abstract

CRISPR-Cas9 screening libraries have arisen as a powerful tool to identify protein-coding (pc) and non-coding genes playing a role along different processes. In particular, the usage of a nuclease active Cas9 coupled to a single gRNA has proven to efficiently impair the expression of pc-genes by generating deleterious frameshifts. Here, we first demonstrate that targeting the same gene simultaneously with two guide RNAs (paired guide RNAs, pgRNAs) synergistically enhances the capacity of the CRISPR-Cas9 system to knock out pc-genes. We next design a library to target, in parallel, pc-genes and lncRNAs known to change expression during the transdifferentiation from pre-B cells to macrophages. We show that this system is able to identify known players in this process, and also predicts 26 potential novel ones, of which we select four (two pc-genes and two lncRNAs) for deeper characterization. Our results suggest that in the case of the candidate lncRNAs, their impact in transdifferentiation may be actually mediated by enhancer regions at the targeted loci, rather than by the lncRNA transcripts themselves. The CRISPR-Cas9 coupled to a pgRNAs system is, therefore, a suitable tool to simultaneously target pc-genes and lncRNAs for genomic perturbation assays.

**Supplementary Information:**

The online version contains supplementary material available at 10.1186/s12864-022-08612-7.

## Background

CRISPR-Cas9 library screening has become a powerful technique to identify genes, both protein coding genes (pc-genes) and long non-coding RNAs (lncRNAs), that play functional roles in cellular processes, such as cell differentiation or cancer progression. Actually, candidates identified in this type of screens have been proposed as potential therapeutic targets, reviewed in [1].

CRISPR screens targeting protein-coding genes (pc-genes) are mainly based on single guide RNAs (sgRNAs) libraries that induce indel mutations in the target genes, leading to frameshifts and, consequently, loss of protein function, reviewed in [2–5]. However, a recent study revealed residual protein activity for some targets after induction of frameshift mutations, leaving room for improvement [6]. This sgRNA strategy is particularly ineffective when targeting long-non coding RNAs (lncRNAs), as point mutations or small indels will not affect the activity of the transcript, in most cases [7].

To overcome this limitation, two different approaches have been followed: CRISPR deletion (CRISPR-del)—also known as CRISPR-ko—and CRISPR interference (CRISPRi), reviewed in [1, 7, 8]. CRISPR-del requires the use of the Cas9, or other nucleases, together with paired guide RNAs (pgRNAs) to induce deletions at the genomic level, either of the entire locus—with the risk of also deleting some overlapping genomic elements [9, 10]—or of the region surrounding the transcription start site (TSS), impairing, in this way, the initiation of transcription [11–13]. Following this approach, we and others have developed different methods involving two guide RNAs cloned in the same vector, both targeting the same gene and simultaneously delivered in the same cell [11, 14–16]. In particular, with the DECKO (Double Excision CRISPR Knockout) system we were able to efficiently promote deletions of up to 3 Kb in cells expressing the Cas9 nuclease [11]. As an alternative approach, CRISPRi requires the use of a catalytically inactive Cas (dCas) [17] fused to a repressor domain such as Krüppel-associated box (KRAB) [18] together with a single guide RNA (sgRNA). The delivery of both components into the same cell promotes the repression of the target gene, either coding or non-coding. CRISPRi knockdown can be tunable and reversible, making it more appropriate for some particular applications than CRISPR-del [19]. Nevertheless, the larger size of Cas9 protein fused to a repressing domain can impair its delivery to target cells and make the system less efficient [20]. Besides, CRISPRi is not suitable for targeting bidirectional promoters, as the repressive domain would induce the unspecific knockdown of all proximal TSSs [21, 22], while CRISPR-del can be directed to target promoter regions with adjacent TSS with high specificity [23].

While the function of many lncRNAs remains unknown, few CRISPR “loss of function” screens have been performed targeting this gene class specifically [13, 21, 24], or targeting a combination of pc-genes and lncRNAs [15]. These studies highlight the high cell type specificity of many lncRNAs, which is especially convenient for tissue-specific targeted therapies.

Cell transdifferentiation is the process by which differentiated somatic cells are reprogrammed into other cell types without transitioning through a pluripotent state. This is of special interest for the development of novel therapies, reviewed in [25]. Thus, the study of the genetic basis and the molecular changes occurring during transdifferentiation is essential to understand and control the conversion between cell types.

One powerful transdifferentiation model is the conversion of human B-cell precursor leukemia cells (BLaER1) to macrophages [26]. BLaER1 pre-B cells are able to transdifferentiate into macrophages upon induction in a process that lasts 7 days. These pre-B cells stably express the hematopoietic transcription factor ratCEBPa fused to an estrogen receptor (ER) hormone binding domain. When β-estradiol is added to the medium, it binds to CEBPaER and allows its translocation into the nucleus, where it induces the transcriptional program leading to macrophage morphology and function [26]. During the transdifferentiation process, it is crucial to shut down the B-cell related expression program and activate the macrophage related one. However, the means by which CEBPa orchestrates the transdifferentiation process remains elusive.

With the goal of discovering pc-genes and lncRNAs essential for the transition from B-cell to macrophage, and taking advantage of available RNA-Seq data produced along the transdifferentiation process [27], we have used the DECKO system [11] with a combined library of paired guide RNAs (pgRNAs), targeting simultaneously 166 lncRNAs and 874 pc-genes upregulated along the transdifferentiation process. Towards that end, we have extended the CRISPETa bioinformatics pipeline [12] to design optimal pairs of sgRNAs for deletion of genomic regions including both pc-genes and lncRNAs. We have observed that targeting pc-genes with two gRNAs synergistically enhances the CRISPR knockout efficiency. The results from our screen suggest that the transdifferentiation from B-cell into macrophage is very robust, and very few genes are able to perturb the progression of the process. Still, out of the targeted genes, we identified 26 candidate genes potentially delaying the transdifferentiation, seven of which were individually validated. Among them, two pc-genes, *FURIN* and *NFE2*, and two lncRNAs, *LINC02432* and *MIR3945HG*, were further interrogated at genomic and transcriptomic level, confirming the efficiency of the pgRNA DECKO system in knocking out protein and lncRNA expression. The fact that some of the identified candidates have been previously associated with blood differentiation and response to infection [28–31] confirms that this system is suitable to specifically uncover both pc-genes and lncRNAs involved in such processes, while it provides new potential candidates for further characterization. In the case of the lncRNAs, the knock down experiments indicated that the lncRNAs transcripts may not be directly involved in the regulation of transdifferentiation, but the impact of the CRISPR-Cas9 interference in the process may be mediated by enhancer regions at the targeted loci.

## Results

### Cellular model and target selection

**Fig. 1:**
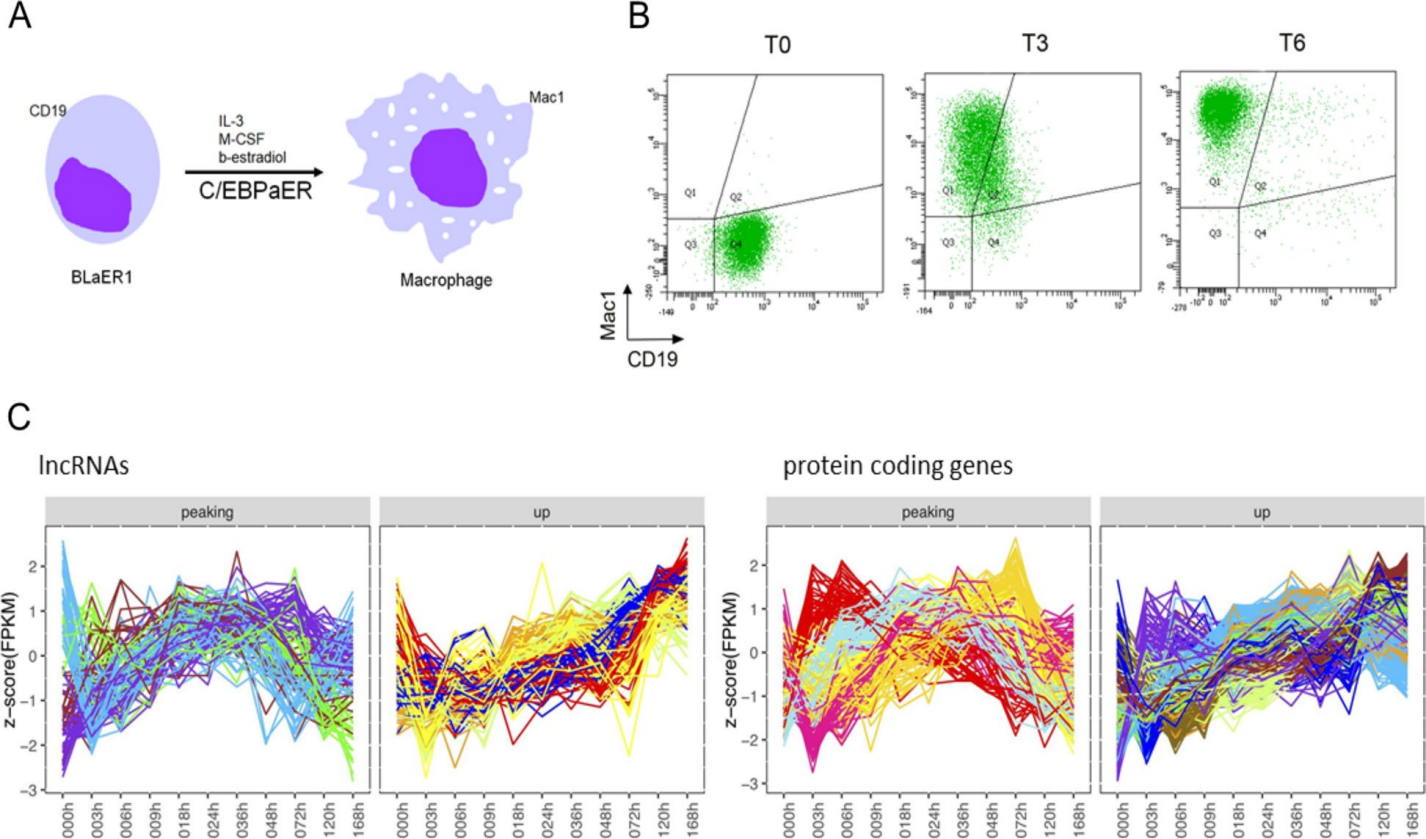
Cellular model and targets selection. **A** Transdifferentiation of BLaER1 pre-B cells into macrophages is accompanied by a dynamic transcriptomic remodeling of the cells. BLaER1 lymphocytes transdifferentiate into functional macrophages in the presence of Interleukin 3 (IL-3) and Macrophage colony-stimulating factor (M-CSF) upon β-estradiol induced release of CEBPaER to the nucleus. **B** Flow cytometry analysis of cell surface markers at T0, T3 (3 days) and T6 (6 days) after induced transdifferentiation in the BLaER1-Cas9 cell line. During the process, BLaER1 cells progressively lose the CD19 (B-cell marker staining -X-axis-) and gain the Mac1 (macrophage marker staining -Y-axis-). **C** Merged k-means clustered expression profiles (color code) of peaking and upregulated genes during transdifferentiation: 16 initial clusters of lncRNA (*n* = 174) and 36 initial clusters of protein coding genes (*n* = 939). FPKM values were log10 transformed before the normalization to z-score. Each line shows the expression pattern of a gene along transdifferentiation. The color corresponds to the k-means cluster to which the gene belongs (see also Supplementary Fig. S1 and S2)

BLaER1 is a leukemia B cell line able to transdifferentiate into macrophages through the stable expression of the transcription factor ratCEBPa fused to an estrogen receptor hormone binding domain [26] (Fig. 1A). During transdifferentiation, the changes in the cell identity can be monitored by flow cytometry through the tracking of specific cell surface markers. For example, the expression of the B cell marker CD19 decreases during the process until disappearing, not being detected in the transdifferentiated macrophages, whereas Mac1, a macrophage surface marker, starts appearing in the transdifferentiating B-cells at 36 h after induction and its detection is maximized at the end of the process (Fig. 1B).

To identify coding and non-coding genes that may drive the BLaER1 transdifferentiation process, we analyzed available RNA-Seq data at 12 time points along the seven days the process lasts, in two biological replicates [27]. We identified 488 lncRNAs and 3,627 pc-genes with expression values above 1 FPKM in at least one time point as well as expression changes higher than twofold for lncRNAs and fourfold for pc-genes (see Methods). We clustered the 4,115 genes with k-means into 16 lncRNA and 36 protein coding clusters (Supplementary Fig. S1 and S2). After visual inspection, genes from all clusters showing upregulated and peaking profiles were selected as candidates to be involved in the transdifferentiation process, comprising in total 174 lncRNAs and 939 pc-genes (Fig. 1C). For both lncRNAs and pc-genes, upregulated genes show higher expression than peaking genes, which peak at about 36 h (Supplementary Fig. S3).

### A CRISPR knockout library targeting simultaneously non-coding and protein coding genes

**Fig. 2:**
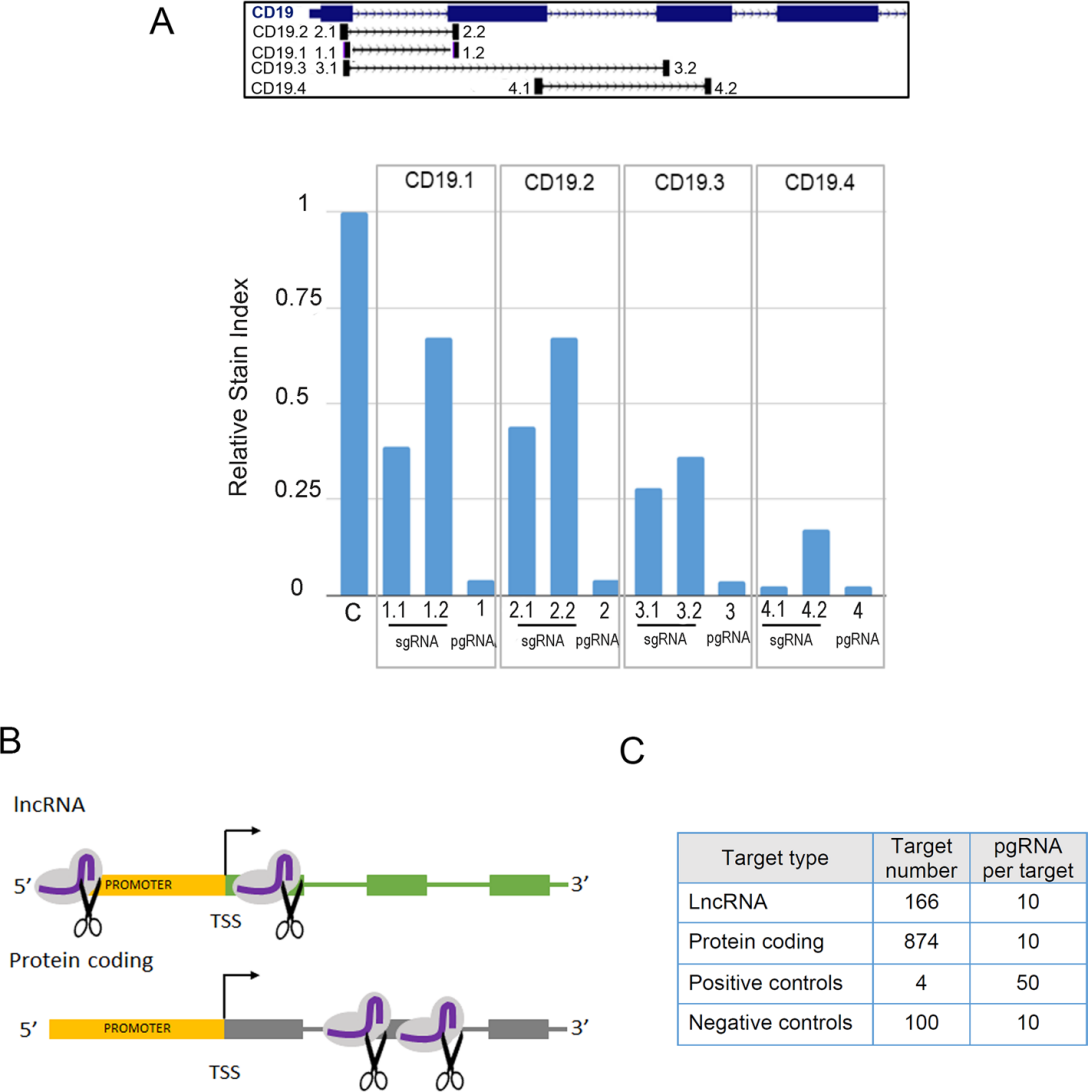
pgRNA CRISPR library for lncRNA and pc-genes. **A** (Upper panel) Diagram of the CD19 gene indicating the target sequence of CD19 pgRNAs (sgRNA1 and sgRNA2, from left to right). (Lower panel) Flow cytometry analysis of fluorescence intensity of the CD19 protein in BLaER1-Cas9 cells infected with sgRNAs and pgRNAs. The relative Stain Index of the different infected cells compared to the maximum expression level of CD19 in control cells (BLaER1-Cas9 cells infected with pDECKO-GFP [11]) is represented. CD19 expression is reduced between 30 and 95% upon infection of sgRNAs. The infection of pgRNAs induces a consistent reduction of CD19 signal up to 95% with all pgRNAs tested. **B** Schematic diagram showing the position of pgRNAs targeting lncRNAs (targeting the promoter and the transcription start site) and pc-genes (targeting coding exons). **C** CRISPR library composition (number of targets of each biotype and pgRNA pairs designed per target)

We first asked whether a pgRNA strategy could yield improved rates of knockout for pc-genes compared to sgRNAs. We, therefore, designed a set of gRNAs against the lymphocyte B surface marker CD19 and infected Cas9 expressing BLaER1 cells with either individual gRNAs or pgRNAs (Supplementary Table S1 and Fig. 2A, upper panel). In order to quantify the efficiency of the knockout, we collected the infected cells and stained them with a fluorescently conjugated anti-CD19 antibody. Single gRNAs caused a 30% to 70% decrease of CD19 immunofluorescence with the only exception of construct CD19-4, whose knockout efficiency is stronger when compared to the negative control (Fig. 2A, lower panel). Although the knockout efficiency of single gRNAs is very variable, the decrease in CD19 signal is enhanced when the cells are infected with any combination of pgRNA (approximately 96% reduction), indicating that the effect of using more than one gRNA per target gene is more than additive.

With the goal of uncovering which peaking and upregulated genes are necessary for the progression of the transdifferentiation, and given the strong synergistic effect observed when targeting pc-genes with two gRNAs, we designed a combined pgRNA CRISPR library targeting simultaneously coding exons of the pc-genes and promoter/TSS regions of the lncRNAs identified above (see Methods, Fig. 2B, Supplementary Fig. S4, and Supplementary Table S2). Using CRISPETa [12], we designed a CRISPR library targeting the 174 lncRNAs. In parallel, we developed a new version of CRISPETa (see Methods) to specifically target protein coding genes, and used it to design pgRNAs targeting the 939 pc-genes selected above at a depth of 10 unique pgRNAs each. According to our on- and off-target filters, we managed to design pgRNAs targeting the TSS of 166 lncRNAs and the ORFs of 874 pc-genes (see Methods, Fig. 2C). As controls, we added pgRNAs targeting pc-genes necessary for transdifferentiation, namely CEBPa (human and rat)—transcription factor used to induce the transdifferentiation [26] -, SPI1—a downstream transcription factor activated by CEBPa needed for macrophage differentiation [32, 33] -, and ITGAM—a subunit of the Mac1 complex used to track macrophage differentiation—(positive controls), and 100 intergenic regions (negative controls) to the library. The CRISPETa output including the pgRNA oligonucleotide sequences can be found in Supplementary Table S2.

### CRISPR-Cas9 screening for genes involved in transdifferentiation

**Fig. 3:**
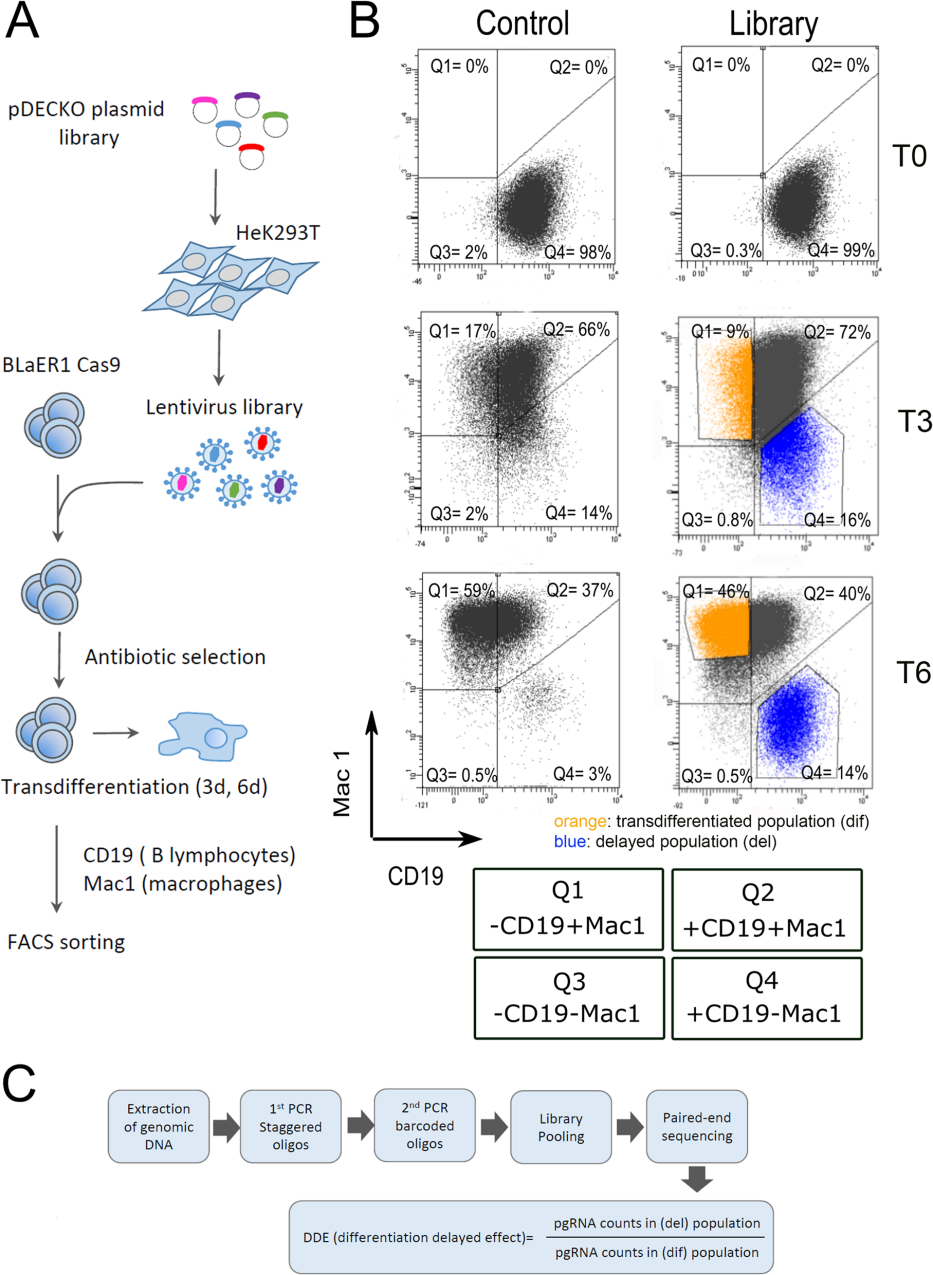
CRISPR-Cas9 screening in BLaER cells**.** **A** Workflow of the CRISPR screening experiment. The pDECKO plasmid library was transfected into HeK293T cells to obtain a library of lentivirus. BLaER1-Cas9 cells were infected at a low multiplicity of infection and double selected with antibiotics (Blasticidin and Puromycin) for 20 days. The infected cells were induced for transdifferentiation into macrophages for 3 days (T3) and 6 days (T6). Cells were labeled with antibodies against cell surface markers: CD19 (for B-lymphocytes) and Mac1 (for macrophages). Transdifferentiation status was assessed by flow cytometry. Transdifferentiated and delayed populations were isolated by Fluorescence-Activated Cell Sorting (FACS). **B** Flow cytometry analysis of BLaER1-Cas9 cells infected with the pDECKO_non-targeting control (left panels) and with the pDECKO_CRISPR-library (right panels) at T0, T3 and T6 of transdifferentiation. CD19 antibody, conjugated with BV510 fluorophore, was used to identify B-cells and Mac1 antibody, conjugated with PE-Cy7 fluorophore, was used to identify macrophages. Quadrants are as follows: Q1 (macrophage-like cells with presence of Mac1 and absence of CD19 surface markers); Q2 (transition cells with the presence of Mac1 and CD19); Q3 (background and not stained cells, negative for Mac1 and CD19); Q4 (lymphocyte B-like cells with the presence of CD19 and absence of Mac1 surface markers). The percentage of cells in each of the 4 quadrants is shown. The fraction of sorted cells showing a delay of transdifferentiation (“delayed” fraction) is marked in blue (gate P4), and sorted cells that differentiate at a normal pace (“differentiated” fraction) are marked in orange (gate P5). See also Supplementary Fig. S5. **C** Workflow for processing the sorted cell populations for deep sequencing. Genomic DNA of sorted cells was extracted and PCR amplified in two steps. For the first PCR, specific staggered primers were used to amplify the integrated fragment which contains the pgRNAs. For the second PCR, Illumina barcoded primers were used to pool different samples (see also Supplementary Fig. S4). Samples were sequenced by 150 bp paired-end Illumina sequencing. DDE (differentiation delayed effect) was calculated as the ratio of pgRNA counts in the delayed population versus the counts in the transdifferentiated population

To identify the genes involved in the transdifferentiation from B cells to macrophages, BLaER1-Cas9 cells were infected with the combined library at low multiplicity of infection (Fig. 3A). In parallel, a plasmid containing non-targeting pgRNAs was transduced as negative control. Cells were collected at 3 days (T3) and 6 days (T6) after induction, and their transdifferentiation status was tracked by flow cytometry with B-cell and macrophage-specific markers (Fig. 3B and Supplementary Fig. S5). We expected to find pgRNAs targeting genes required for transdifferentiation in the “delayed” cell population, which progresses at a slower rate compared to the control cells (quadrant Q4, corresponding to undifferentiated cells, in Fig. 3B compare left -control- vs right -library- panels and Supplementary Fig. S5).

Whereas the library infected cells only show a mild delay in comparison to the negative control at T3 of transdifferentiation (16% vs 14% in Q4, respectively) (Fig. 3B and Supplementary Fig. S5), the difference is much stronger at T6 (14% vs 3% in Q4, respectively). To identify the pgRNAs responsible for the delay of transdifferentiation, the delayed (blue gates) and the differentiating (orange gates) populations were recovered by fluorescence-activated cell sorting (FACS) at T3 and T6 after transdifferentiation induction, and the integrated pgRNAs were sequenced (see the workflow on Fig. 3C and see Methods).

### Identification of lncRNAs and protein coding genes involved in transdifferentiation delay

On average, 25 million reads were sequenced for each isolated population. We implemented a bioinformatics protocol to analyze and quantify these reads (Supplementary Fig. S6, see Methods). The distribution of pgRNAs of the original library after cloning showed a similar profile to the distribution of pgRNAs identified upon transdifferentiation induction (T0), demonstrating that all pgRNAs in the initial library were represented in the screening. However, during the course of the transdifferentiation (T3 and T6), a small fraction of guide pairs became enriched while many others were depleted (Supplementary Fig. S7).

To identify the pgRNAs enriched in each subpopulation of cells, we defined the differentiation delaying effect (DDE) as the ratio of counts of a given pgRNA from the delayed subpopulation (del) divided by the counts from the transdifferentiated population (dif) (Fig. 3C). Thus, larger DDE values would represent genes required for the correct transdifferentiation. DDE was computed independently for the two replicates at T3 and T6 after transdifferentiation.

**Fig. 4:**
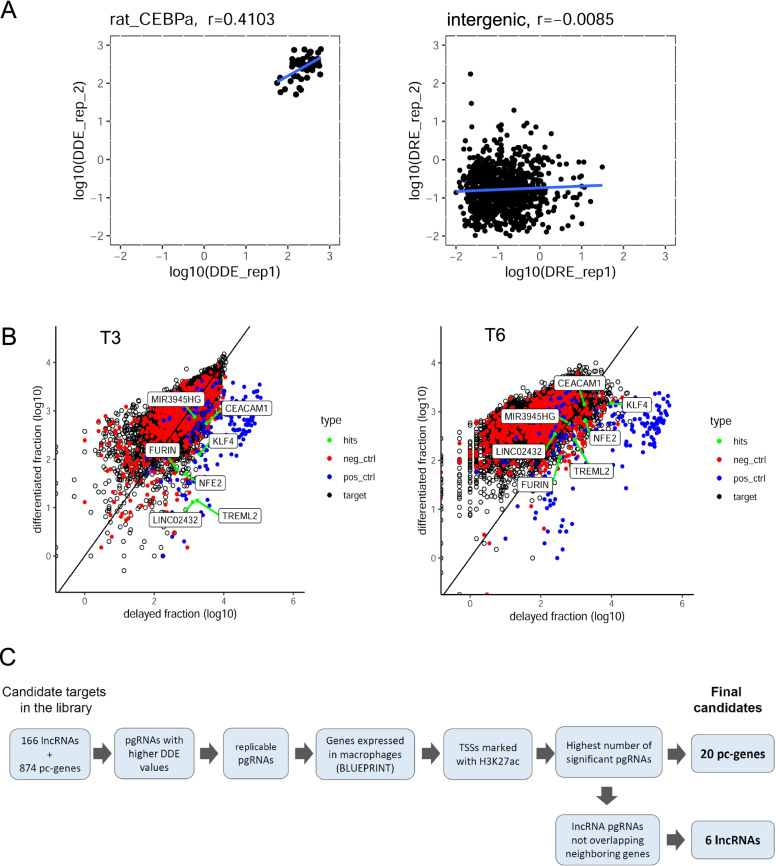
Identification of lncRNAs and protein coding genes involved in transdifferentiation. **A** Correlation between replicates of the differentiation delaying effect (DDE, ratio of reads from delayed versus transdifferentiated fraction) observed per pgRNA of ratCEBPa (left panel) and intergenic negative controls (right panel) after 6 days (T6) of transdifferentiation. Each dot represents a different pgRNA. Spearman correlation values are stated above. The DDE values of CEBPa pgRNAs are very large and show a positive correlation between replicates, whereas intergenic pgRNAs do not show reproducible DDE values between replicates. **B** Scatterplot of log10 transformed counts in delayed versus differentiated fractions at T3 and T6 after induction of transdifferentiation. Each dot represents a different pgRNA. pgRNAs targeting positive controls are depicted in blue, intergenic pgRNAs in red, screened candidates in black, and pgRNAs of selected candidates showing high average DDE score in green (merged counts of both replicates). **C** Decision tree followed to identify candidate genes, from the CRISPR-Cas9 screening, involved in the transdifferentiation process. From the original list of 1,040 pc-genes and lncRNAs, we ended up with a set of seven candidates to undergo further validation

We first assessed whether the DDE score could distinguish between positive and negative controls. Indeed, ratCEBPa pgRNAs show reproducible large DDE values that correlate between replicates (for all tested sets of pgRNAs, Fig. 4A left panel). The values for all the intergenic pgRNAs are much lower (Fig. 4A right panel), showing no reproducibility between replicates.

We next plotted the count distribution of the pgRNAs detected in the delayed fraction against the differentiated fraction (Fig. 4B). Confirming the efficiency of the methodology, pgRNAs targeting positive controls (blue) show a higher enrichment in the delayed fraction compared to the differentiated one, whereas negative control pgRNAs (red) move from the diagonal at T3 to the differentiated fraction at T6. Although, at T3, the bulk of pgRNAs targeting candidate genes are centered around the diagonal, a number of pgRNAs show enrichment in the delayed population, which is attenuated with an overall shift of guides towards the differentiated fraction at T6 of transdifferentiation.

In order to identify potential target genes affecting the transdifferentiation process, we selected all pgRNAs with DDE values in the highest decile (at T3 DDE > 1.89, at T6 DDE > 0.44, mean of both replicates) (Supplementary Fig. S8A). Besides that, for T3 and T6 separately, we required potential targets to have at least two identical pgRNA pairs in the upper decile for both biological replicates. Following this criteria, 18 lncRNAs and 86 pc-genes were selected at T3 and 50 lncRNAs and 135 pc-genes at the T6 time point. The union of candidates from both time points resulted in a total of 64 lncRNAs and 191 pc-genes (Supplementary Table S3). Comparing the distribution of the DDE values of all the pgRNAs corresponding to the selected target genes against positive and negative controls revealed significant differences between them, especially at T3 after induction, when both lncRNA and protein coding targets show significantly higher DDE values than the negative intergenic controls (Supplementary Fig. S8B).

To further narrow down the candidate list for individual validations, we applied additional criteria (Fig. 4C). First, we checked the consistency of the expression of the candidates along the hematopoietic tree (Blueprint RNA-Seq quantifications from the Blueprint Dataportal http://dcc.blueprint-epigenome.eu/) and discarded candidates with either no expression in B-cells/macrophages or unexpected relative expression, e.g. significantly lower expression in macrophages than in B-cells. Second, we selected candidates that showed H3K27ac marking at the TSS along seven ENCODE cell lines, a mark that has been related to both active promoters and enhancers [34, 35]. Third, we selected the candidate genes with the highest number of pgRNAs significantly enriched in the delayed population in comparison to the differentiated one. Finally, for lncRNAs, we further verified that the pgRNAs targeting the promoter region did not overlap any other neighboring gene. Considering all these criteria, we ended up with 6 lncRNAs and 20 pc-genes as candidates to be involved in transdifferentiation (Supplementary Table S3). From them, the top two lncRNAs -*LINC02432 and MIR3945HG*—and five protein coding genes—*FURIN, NFE2, KLF4, TREML2* and *CEACAM1* -, following the aforementioned criteria, were selected as the targets with the highest potential to impact transdifferentiation efficiency (RNA expression profiles of these candidates along transdifferentiation can be found in Supplementary Table S4).

**Fig. 5:**
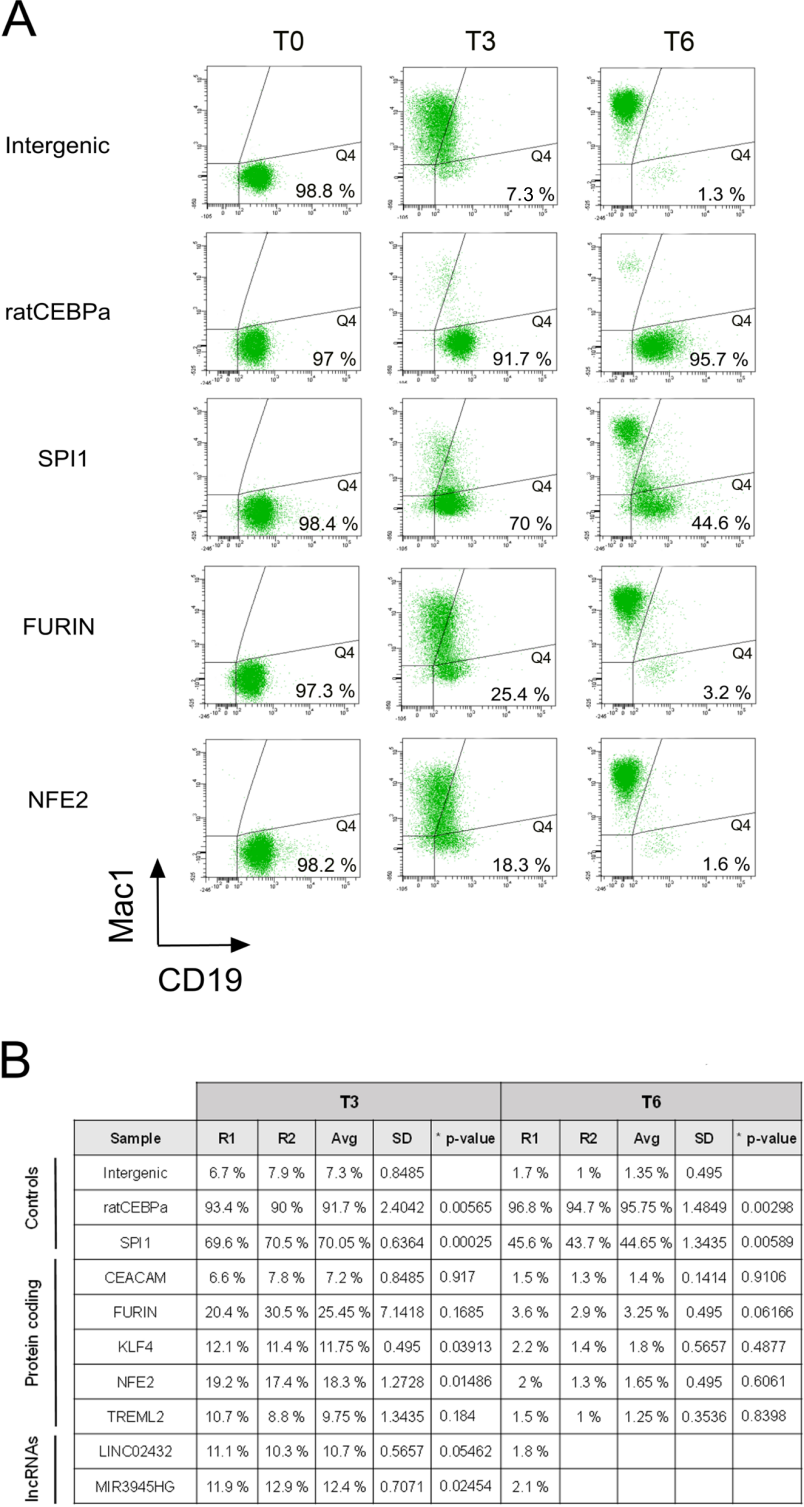
Individual target validation by flow cytometry. Flow cytometry analysis of control and candidate pgRNAs at T0, T3 and T6 after induction of transdifferentiation. **A** Flow cytometry plots of intergenic negative control, two positive controls targeting *ratCEBPa* and *SPI1*, and two protein coding targets *FURIN* and *NFE2*. CD19 B-cell marker is represented on the X-axis and Mac1 macrophage marker is represented on the Y-axis. Cells that do not undergo transdifferentiation remain in the Q4 quadrant (positive for CD19 -X-axis- and negative for Mac1 -Y-axis) (the percentages of cells in this quadrant are shown). **B** Percentage of cells with delayed transdifferentiation (Q4 quadrant) observed in controls and individually validated candidates for two biological replicates (R1 and R2) at T3 and T6 after induction of transdifferentiation. For lncRNAs *LINC02432* and *MIR3945HG* we only have data for one biological replicate at T6. Average (Avg) and standard deviation (SD) between replicates, and two-tailed p-values (comparing the delayed population from each individual target and the intergenic negative control) are also shown. Most of the selected candidates show significant delay compared to the intergenic negative control at T3. Although the value observed for FURIN is not statistically significant, the magnitude of the delay indicates that it is a strong candidate to perform further validations

We next wanted to assess if the candidate genes identified in the CRISPR screening did, indeed, play a role during the transdifferentiation process. Thus, for each control and target gene, we selected pgRNAs showing high and reproducible enrichment in the delayed population compared to the differentiated one (Supplementary Table S1). First, we individually validated the delay of positive control pgRNAs against *CEBPa* and *SPI1* compared to the intergenic negative ones. The delay effect was measured by tracking the expression of CD19 and Mac1 at T3 and T6 after induction (delayed cells are represented in the Q4 quadrant, Fig. 5A). Indeed, we observed a strong delay for cells expressing pgRNA against *CEBPa* and *SPI1* compared to intergenic regions both at T3 and T6 (Fig. 5) indicating that the efficiency of the pgRNA knockout is very high when targeting protein coding genes, which is consistent with the high efficiency observed when knocking out CD19 (Fig. 2A). To further confirm the knockout of *CEBPa* and *SPI1*, we performed additional validations at the genomic level (Supplementary Fig. S9). In both cases, all clones tested show rearrangements surrounding both gRNA homology regions, highlighting, again, the high efficiency of the pDECKO system in knocking out pc-genes. Note that none of the tested clones shows the long deletion expected if both pgRNAs induced the Cas9 cut. Still, targeting only one of the two regions can generate a frameshift, resulting in a non-functional protein. In the case of CEBPa, we also verified the decrease at the protein level by western blot (Supplementary Fig. S10).

We next assessed the effect on transdifferentiation efficiency when knocking out the selected candidate genes. Cells infected with pgRNAs against the two lncRNAs (*LINC02432* and *MIR3945HG*) show some initial delay in transdifferentiation (10–12% at T3), whereas they seem to fully recover at T6, showing delays comparable to the negative intergenic controls (Fig. 5B and Supplementary Fig. S11 A-B). For pc-genes, the knockout of *FURIN* and *NFE2* has the strongest delaying effect on transdifferentiation (25% and 18% at T3 respectively, Fig. 5). For the remaining genes tested, undifferentiated cells range between 10 and 12%, except for *CEACAM1*, which shows a delay comparable to the intergenic negative control (Fig. 5B and Supplementary Fig. S11 C-G).

### Individual validation of lncRNAs involved in the transdifferentiation process

To further validate the role of the two candidate lncRNAs in transdifferentiation, we assessed whether the CRISPR-Cas9 was able to efficiently induce a deletion at the promoter region of these genes. Thus, we isolated by FACS the cell populations corresponding to the delayed fractions from the individual validations above and amplified and sequenced the region surrounding their TSS. Indeed, we could validate the double cut, as well as diverse rearrangements with multiple indels, in the clones from the *LINC02432* targeted cells tested (Supplementary Fig. S12 A-B, Supplementary Fig. S13A). In the case of the *MIR3945HG*, however, we were not able to confirm the deletion at the genomic level (Supplementary Fig. S13B).

To distinguish if the role of these lncRNAs on transdifferentiation was mediated by the RNAs themselves or by a putative enhancer effect of the DNA regions transcribing the lncRNAs, we designed LNA GapmeRs against the two lncRNAs targeting the same isoforms as were depleted by CRISPR. Although the expression of both lncRNAs was impaired upon GapmeR treatment, specially for *LINC02432*, we did not observe any transdifferentiation delay (Supplementary Fig. S12 C-D). This suggests that the impact of the deletion of these two lncRNAs on the process is likely due to the disruption of a possible enhancer activity of the deleted genomic sequence. Consistent with this hypothesis, both loci are enriched in H3K27ac, a mark associated with active enhancers [34], and this enrichment increases upon induction of the process (Supplementary Fig. S14) [27, 36].

Trying to untangle the possible role of *LINC02432* and *MIR3945HG* loci as enhancer regions, we took advantage of publicly available data on enhancer/promoter pairs on B cell and macrophage lineages (Activity By Contact or ABC dataset, [37]) to identify their interacting regions. For the *LINC02432*, we identified several regions surrounding the lncRNA TSS that show significant interactions with the nearby *ZNF330* locus, located 90 Kb away. Although none of these regions overlaps with the depleted region in the CRISPR-Cas9 validation, we cannot discard the possibility that the deletion of the lncRNA TSS may impact the transdifferentiation process through these close interactions with *ZNF330* and/or other pc-genes. Likewise, we performed a similar analysis with *MIR3945HG*. In this case, we found that the region targeted around the TSS of the *MIR3945HG* interacts with several neighboring genes. Among them, we found *ACSL1*, located 30 Kb downstream of the lncRNA (Supplementary Fig. S14). *ACSL1* has been involved in promoting inflammation in monocyte-derived macrophages [38]. *ACSL1* is actually up-regulated along transdifferentiation, and its expression shows a correlation of ~ 0.86 with the expression of the lncRNA. *MIR3945HG* also interacts with another pc-gene, *ANKRD37* (Supplementary Fig. S14), showing also a strong positive expression correlation (~ 0.88). *ANKRD37* has no known role on macrophage activity and has been related to trophoblast migration and preeclampsia risk during pregnancy [39]. The strong correlation of the expression of *MIR3945HG* with these two genes is suggestive of co-regulation.

We finally analyzed recently released data on active/silent compartments, A/B compartments, during BLaER1 cells transdifferentiation [40]. We found that *MIR3945HG* stays in the A compartment during the process. *LINC02432,* instead, is found in the B, inactive, compartment in pre-B cells, but at 72 h it turns into A compartment, turning inactive again later in transdifferentiation, reflecting the peaking expression profile of the gene (Supplementary Table S4). The interactions observed for both *LINC02432* and *MIR3945HG* with neighboring pc-genes and the presence of these loci in active compartments further support the implication of these regions in the transdifferentiation process, likely through the regulation of distal target genes.

### Individual validation of protein coding genes involved in the transdifferentiation process

**Fig. 6:**
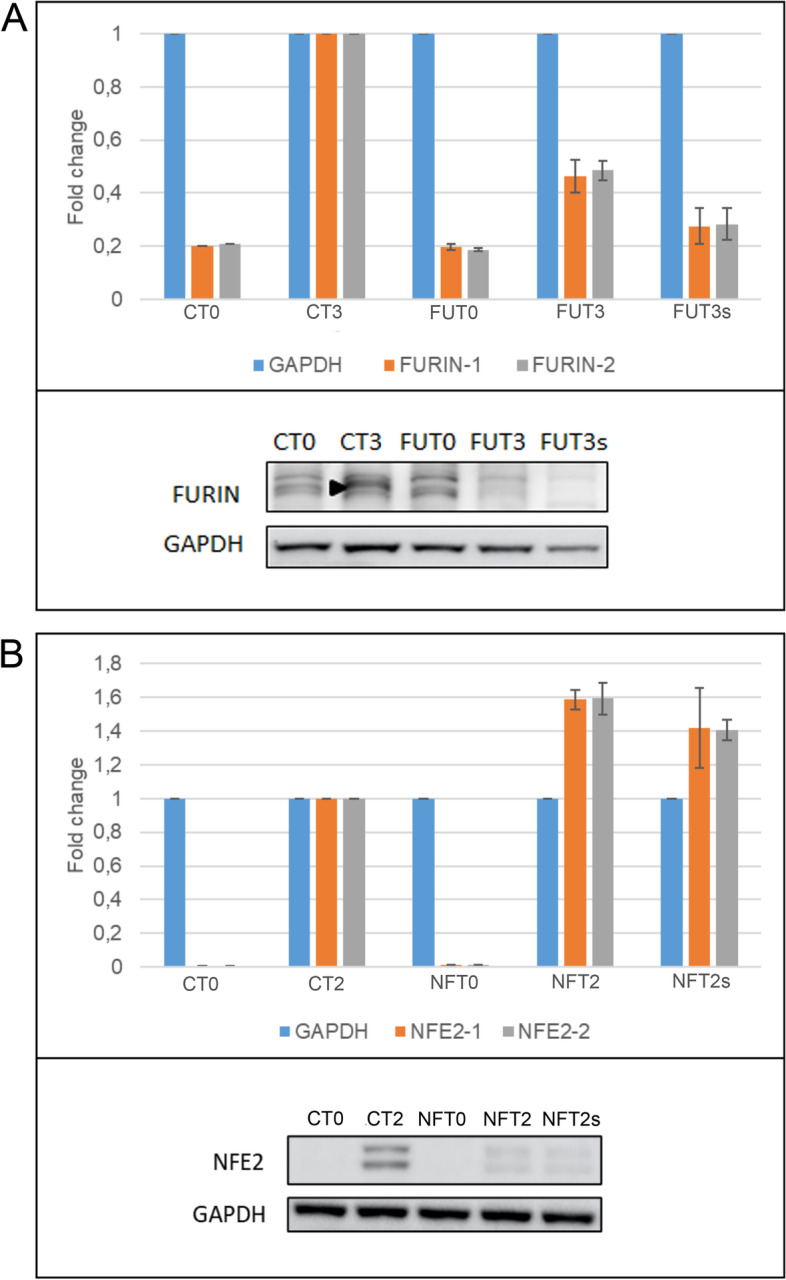
FURIN and NFE2 expression after CRISPR edition. **A** FURIN RNA and protein expression. Cells were collected at T0 (before induction) and T3 (3 days after transdifferentiation induction). (CT0) and (CT3) negative control pDECKO-Intergenic at T0 and T3 respectively, (FUT0) and (FUT3) pDECKO-FURIN at T0 and T3, (FUT3s) pDECKO-FURIN at T3 and sorted from gate P4 (delayed population). Upper panel, qRT-PCR to check the expression of FURIN using two different sets of primers. Results are normalized to GAPDH and the fold change is calculated relative to the expression of cells infected with pDECKO-intergenic pgRNA at T3. The expression of FURIN decreases in cells infected with FURIN pgRNAs, especially in the delayed subpopulation (FUT3s). Bottom panel, western blot to assess the levels of the FURIN protein in BLaER1-Cas9 infected cells. Anti-FURIN antibodies recognize a band (marked with an arrowhead), the signal of which increases at T3, in line with RNA-Seq data (Supplementary Table S4). The FURIN band is not detectable in the pDECKO-FURIN infected cells (FUT3 and FUT3s). Uncropped blots are shown in Supplementary Fig. S16A. **B** NFE2 RNA and protein expression. (CT0) and (CT2) negative control pDECKO-Intergenic at T0 (before induction) and T2 (2 days after transdifferentiation induction) respectively, (NFT0) and (NFT2) pDECKO-NFE2 at T0 and T2, (NFT2s) pDECKO-NFE2 at T2 and sorted from gate P4 (delayed population). Upper panel, qRT-PCR to check the expression of NFE2 using 2 different sets of primers. Results are normalized to GAPDH and the fold change is calculated relative to the expression of cells infected with pDECKO-intergenic T2. NFE2 expression in NFE2 pgRNA targeted cells is higher than in intergenic control cells (NFT2 and NFT2s compared to CT2). Bottom panel, western blot to check the protein levels of NFE2 in BLaER1-Cas9 infected cells. Anti-NFE2 antibodies detect two bands, the signal of which increases at T2 (CT2 compared to CT0). These two bands are strongly reduced in NFE2 targeted populations (NFT2 and NFT2s compared to CT2). Uncropped blots are shown in Supplementary Fig. S16B

Regarding the pc-gene candidates, we characterized *FURIN* and *NFE2* at genomic, transcriptomic and protein level. At the genomic level, we could identify different editing events (indels) at the *FURIN* locus (Supplementary Fig. S15 A-B). We observed that all tested clones showed small rearrangements surrounding the two regions targeted by the pgRNAs, inducing frameshift mutations. At transcriptomic level, *FURIN* expression, measured by qRT-PCR, decreases to around 50% in the full population of infected cells at T3 compared to the intergenic negative control (Fig. 6A, FUT3 vs. CT3, respectively). This decrease reaches 70% when only the delayed population is measured (Fig. 6A, FUT3s). Although we do not expect the deletion of an internal part of the gene to affect transcript abundance, we hypothesize that the lack of functional protein may cause the degradation of the transcript by nonsense mediated decay. We also observed that the decreased gene expression has an impact at protein level, as the FURIN protein is not detectable by western blot in CRISPR-Cas9 edited cells at T3, compared to the intergenic control, where a specific band is detected (Fig. 6A).

For *NFE2*, we also identified small indels at the genomic level (Supplementary Fig. 15 C-D). The fact that not all the clones show mutations at the two regions highlights the advantage of using paired gRNAs to increase the knockout efficiency when targeting pc-genes. In this case, RNA expression analysis showed that, for CRISPR-Cas9 edited cells, *NFE2* expression increases compared to negative control cells after 2 days of transdifferentiation (T2) (Fig. 6B). Protein levels, in contrast, appear to decrease at this time point (Fig. 6B). We hypothesize that this contrasting pattern between *NFE2* transcript and protein expression could potentially be explained by the production of non-functional protein promoting the continuous overexpression of the gene, in an attempt to overcome the lack of functional NFE2 protein.

## Discussion

Along this manuscript, we have described the use of the CRISPR-Cas9 technology to identify genes, both lncRNAs and protein coding, involved in the transdifferentiation from pre-B cells into macrophages. With this goal, we have designed a library targeting simultaneously the TSS of lncRNAs and the coding region of pc-genes. We think that such a combined library is a suitable approach to identify large numbers of target candidates independently of their biotype. Besides, our library design can be customized to target not only genes but also genomic regions putatively involved in dynamic processes, for instance enhancers or chromatin insulators. In particular, the pgRNA library generated in this work represents a convenient resource to perform knockout screens in other models, such as macrophage differentiation, as many of the potential targets involved in transdifferentiation may also be candidates in these other systems.

We had previously demonstrated that the DECKO system is able to efficiently induce deletions of up to 3 Kb around the TSS of lncRNAs, and that these deletions impaired gene expression [11]. Here, we further wanted to assess whether using paired gRNAs would significantly increase the efficiency of the knockout of protein coding genes. Indeed, we found that targeting the CD19 B lymphocyte marker with paired gRNAs is more efficient than targeting it with a single gRNA. Actually, the decrease in protein expression after pgRNA infection is more than additive, meaning that the usage of two gRNAs synergistically enhances the efficiency of the CRISPR system due to a combination of indels in one or both gRNA target sites and/or deletions of the full region. Consistent with this strong effect, we have also observed a strong transdifferentiation delay in cells infected with pgRNAs targeting *CEBPa* and *SPI1*. We hypothesize that the different efficiency observed between *CEBPa* and *SPI1* knockdown may be due to differences in gene copy number. Although BLaER1 cells show high levels of CEBPa compared to other clones [26], we speculate that *ratCEBPa* is only present at one copy per cell, as more than 90% of cells show a delay of transdifferentiation, indicating that almost all cells have been knocked out. In contrast, pgRNAs against *SPI1* are expected to target the two endogenous copies of the gene; thus, the fact that 45% of cells targeted with pgRNAs against *SPI1* show delayed transdifferentiation suggests that in around 50% of cells the two *SPI1* copies have been efficiently knocked out. Given the high specificity of gRNAs on leading the Cas9 protein to its target regions [41], the usage of pgRNAs could be a particularly appropriate strategy to target polymorphic regions, as a point mutation in the target sequence could result in a dramatic reduction of the targeting/cut efficiency, whereas the usage of pgRNAs could double the chances of genome editing. Overall, and given the high knockout rates observed in all cases, we think that the usage of paired gRNAs represents a convenient approach to target protein coding regions in high throughput screens.

Our results demonstrate the high knockout efficiency of the DECKO system. Nevertheless, we think that the relatively low number of positive candidates identified in the CRISPR-Cas9 screen may obey the strong capability of BLaER1 cells to transdifferentiate. Accordingly, whereas most leukemia and lymphoma cell lines tested were not able to transdifferentiate in an efficient manner, BLaER1 cells are able to efficiently undergo the process, likely due to the constant and high expression levels of transgenic CEBPa [26]. We think that the depletion of non-essential players involved in the transdifferentiation process cannot overcome the severe transcriptomic changes induced in these leukemia B-like cells. Indeed, even when the depletion of FURIN and NFE2 is able to promote an initial delay of transdifferentiation, the targeted cells are able to eventually overcome the lack of these proteins, likely due to the implication of other factors performing the same or similar functions, and transdifferentiate into macrophages after 6 days.

As a result of our screen we identified six lncRNAs and twenty protein coding genes as potential candidates to play a role in the transdifferentiation of B-cells to macrophages. One of the critical points in the library design (especially for lncRNAs) is the correct annotation of TSS [42], which is constantly improved and updated in the new GENCODE releases [43]. The incomplete annotation of the non-coding genes may also influence the correct targeting of these genes, and may partially explain the relatively low validation rate for lncRNAs in this type of screens [13, 21, 24].

Among the validated lncRNAs, on the one side, *LINC02432* was previously identified as an upregulated lincRNA in neuroblastoma cell lines [44]. Here, we have found that the *LINC02432* locus interacts with the *ZNF330* gene in blood-related cell lines. ZNF330 is a zinc-finger protein that has been related to proapoptotic functions in humans [45]. Although *ZNF330* expression does not change along transdifferentiation, we cannot discard that the deletion of the lncRNA TSS is affecting transdifferentiation through its contact with *ZNF330*. Alternatively, the deletion of this region could also affect the expression of other distal genes not identified by the ABC contacts. On the other side, *MIR3945HG*, which is overexpressed in macrophages upon infection with *Mycobacterium tuberculosis*, has been proposed as a candidate marker for the diagnosis of tuberculosis [30]. Here we have also seen that the *MIR3945HG* TSS interacts with several neighboring genes, such as *ACSL1* and *ANKRD37*. In this case, however, the expression of these pc-genes strongly correlates with the expression of the lncRNA, indicating that *MIR3945HG* may indeed participate in the progression of transdifferentiation through the regulation of these pc-genes, maybe through the direct contact between the genomic regions.

The pc-genes validated in this study show a comparable stronger effect. Among them, *FURIN* appears to play the strongest role. This protein is a ubiquitously expressed serine protease enzyme that processes substrates like cytokines, hormones, receptors and growth factors like TGFB1. It controls proliferation and differentiation in many cell types [46] and has been involved in tumor progression, representing an interesting therapeutic target. FURIN has been also related to monocyte/macrophage migration and proliferation, being also an inhibitor of apoptosis [29, 31]. Actually, the expression pattern of *FURIN* suggests that it is involved in the last steps of macrophage lineage determination, consistent with its role in macrophage motility. Another protein with notable effect is the transcription factor NFE2. This factor was found to be essential for regulating erythroid and megakaryocytic maturation and differentiation, but also impacting the renewal of hematopoietic stem cells [28, 47, 48]. Altered NFE2 activity predisposed to leukemic transformation [49] and *NFE2* is overexpressed in the majority of patients with myeloproliferative neoplasms [50]. The other protein coding targets identified in the screen have been also related to blood and/or differentiation functions [51–53]; however, the milder effect observed during transdifferentiation highlights again the robustness of the system.

## Conclusions

All in all, we have designed a CRISPR-Cas9 library to simultaneously target lncRNAs and protein coding genes, and assess their role in B-cell to macrophage transdifferentiation. This screening has led to the identification of a few candidates that could potentially play a role in this process. The low number of candidates and the rapid recovery of the cellular perturbations induced by the CRISPR-Cas9 knockouts indicates, however, that the transdifferentiation of the BLaER1 cells into macrophages is a very stable and robust process. Nevertheless, we have demonstrated that the DECKO libraries are very efficient in promoting both frameshifts and deletions. We believe, therefore, that this is a powerful method for the study of the regulation of dynamic processes, as it is suitable for the efficient knockout of protein coding genes as well as for deletion of small genomic regions, not only lncRNA TSSs, but also putative enhancers and other regulatory regions. Indeed, our results suggest that the two lncRNA loci identified in the CRISPR-Cas9 screen could be actually acting as enhancer regions regulating the expression of other genes.

## Methods

### Target gene selection from transcriptomics data

The selection of target genes was based on RNA-Seq data sampled at 12 time points (0 h, 3 h, 6 h, 9 h, 12 h, 18 h, 24 h, 36 h, 48 h, 72 h, 120 h, 168 h) during transdifferentiation of human BLaER1 cells to macrophages [27]. The RNA-seq data was quantified with GRAPE-nf (https://github.com/guigolab/grape-nf). Read mapping was performed with STAR [54] and gene expression quantification with RSEM [55] using the GENCODE annotation v22 [56]. Two biological replicates were analyzed separately.

The 19,814 pc-genes and 14,855 lncRNAs (union of the following biotypes: processed transcript, 3 prime overlapping ncRNA, sense intronic, antisense, macro lncRNA, lincRNA, non-coding and sense overlapping from GENCODE v22) were filtered for a minimum average expression of at least 1 FPKM for pc-genes (0.1 FPKM for lncRNAs) and at least 4 × fold change for protein pc-genes (2 × fold for lncRNAs) between highest and lowest expression value along the temporal profile. In addition, lncRNAs were required to have a minimum expression of 1 FPKM in at least one time point and to be non overlapping with other genes in a 5 Kb window on the same strand and 50 bp on the opposite strand relative to their TSS. This resulted in 4,804 pc-genes remaining for replicate 1 and 4,552 for replicate 2, and 642 lncRNAs for replicate 1 and 536 for replicate 2. Those genes were clustered separately for each replicate into 36 expression profiles for pc-genes and 16 for lncRNAs with k-means clustering in R. We focused on two types of expression profiles: “peaking profile” (genes that increase their expression level at the beginning of the transdifferentiation process and later on decrease) and “upregulated profile” (genes that are upregulated throughout the process). Pooling those profiles within each replicate and then intersecting between the replicates resulted in a final list of 939 protein-coding and 174 lncRNA candidate genes.

### Paired guide RNA library design

For lncRNAs, CRISPETa [12] was used to target genes’ TSS. For pc-genes, we developed a new version of CRISPETa to target ORFs (code available at https://github.com/Carlospq/CRISPETa_PC). In this case, we first obtained the principal isoform from the APPRIS database [57]. The exonic sequence of this isoform was extracted from the human genome sequence version h19, using the GENCODE annotation v22, and searched for all possible protospacers (20 mers followed by a PAM sequence of NGG). sgRNA were scored using the RuleSet2 algorithm [58] and paired. Pairs were ranked according to: 1) location in the ORF sequence, 2) the pair score calculated as the sum of the two individual sgRNA scores, and 3) the deletion region of the pair (prioritizing those predicted to create an out-of-frame deletion). The first coding exon was preferentially targeted. In case not all designs could be placed at the first coding exon, the window was extended to the second and third exons. For lncRNAs, the region targeted around the TSS was increased stepwise from 500 to 5,000 bp in consecutive runs of CRISPETa until the required number of pgRNAs was designed. Selected pgRNAs for lncRNAs were filtered so as to not overlap pc-genes. In all cases, sgRNAs were filtered to remove possible off-targets using CRISPETa’s pre-computed database with default value [-t 0,0,0,x,x] for the first run and relaxing this cutoff for consecutive runs, as described in [12]. CRISPETa output parameters were adjusted to provide the sequence of the 165 nt oligonucleotide (Insert-1) needed for library cloning using DECKO method [11], which includes the targeting regions of the pgRNAs separated by a cloning site (Supplementary Table S2).

Up to ten pgRNAs were designed per target gene with a minimum distance of 50 bps between any pair of gRNAs. In total, we designed pgRNAs for 166 lncRNAs and 874 pc-genes. In addition, we designed 50 pgRNAs for each ratCEBPa, humanCEBPa, SPI1 and ITGAM positive controls. For negative controls, we designed pgRNAs for 100 intergenic regions, 10 pgRNAs each. We also included some pgRNAs targeting fluorophores (EGFP, mCherry and tdTomato) (see Supplementary Table S2). As a non-targeting negative control for library sorting assays, we used a pgRNA against Firefly luciferase, called “pDECKO-non targeting”.

### Library cloning

A ssDNA library of 12,000 oligos of 165 nt (insert-1) (Supplementary Table S2) was purchased from Twist Biosciences. The library was amplified to obtain dsDNA using emulsion PCR as described in [59], and cloned into pDECKO_mCherry vector ([12], Addgene 78534) following the 2 cloning steps described in [11]. ENDURA electrocompetent cells (Bionova Cientifica) were used to ensure high efficiency transformation and avoid recombination errors. Several transformations were performed in parallel. For the first cloning step (intermediate plasmid), approximately 500,000 bacterial colonies were collected and processed together in a single maxiprep. To eliminate the remaining empty plasmid, we took advantage of the fact that insert-1 (in the intermediate plasmid) contains unique restriction sites (EcoRI and BamHI), which are not present in the original backbone. Digesting the intermediate plasmid resulted in a linear product that could be distinguished from the circular empty backbone and purified in an agarose gel. For the 2nd step of cloning, 50 ng of BsmbI-digested intermediate plasmid was mixed with 1 μl annealed Insert-2 (gRNA1 constant region coupled to an H1 promoter, previously assembled from four oligonucleotides and diluted 1:20) and 1 μl of T4 DNA ligase (Thermo Scientific) and incubated for 4 h at 22ºC (as described in [11]). Several transformations with ENDURA electrocompetent cells were done in parallel. For the 2nd cloning step (final plasmid) more than 100,000 bacterial colonies were collected and processed together in a maxiprep. A scheme of the final plasmid can be found in Supplementary Fig. S4A. The final pooled library was deep sequenced for diversity verification (Supplementary Fig. S7). The library is available at Addgene.org (BLaER1 pgRNA CRISPR library ID 183825).

### Cell culture, library infection and transdifferentiation induction

Human BLaER1 cells [26] were kindly provided by Thomas Graf (CRG, Barcelona) and grown in RPMI medium supplemented with 10% heat-inactivated fetal bovine serum (FBS), 2 mM L-glutamine, and 100 U/ml Penicillin–Streptomycin [26]. BLaER1 cells were first infected with a plasmid containing Cas9 fused to BFP ([12], Addgene 78545), selected for more than 5 days with blasticidin (15 µg/ml) and sorted using a BD FACS Aria instrument at the Flow Cytometry Unit of the Centre for Genomic Regulation. These cells, stably expressing Cas9, were then infected with the pDECKO library. For lentivirus production, we performed 80 co-transfections of HeK293T virus packaging cells (at approximatelly 60–70% confluence on 10 cm dishes) with 3 μg of the pDECKO_mCherry plasmid library (Addgene 183825) and 2.25 μg of the packaging plasmid pVsVg (Addgene 8484) and 750 ng of psPAX2 (Addgene 12260) using Lipofectamine 2000 (Invitrogen), according to manufacturer's protocol. Transfection media was changed on the following day to RPMI. In total, 400 ml of viral supernatant were collected 48 h post transfection, filtered through a cellulose acetate filter, and used for overnight infection of 90 × 10E6 BLaER1-Cas9 cells at a density of 250,000 cells/ml with presence of polybrene (10 μg/ml). The percentage of infection was computed as the number of mCherry positive cells compared to the total number of cells with a Fortessa cell cytometer analyser. Infection rate ranged between 2–4%, ensuring a low multiplicity of infection (less than 1 viral integration per cell) [60]. After 48 h of infection, the cells were double selected with blasticidin (20 μg/ml) and puromycin (2 μg/ml) for 18–19 days. 15 million of the BLaER1-Cas9 library infected cells were induced for transdifferentiation into macrophages by using 100 nM β-estradiol and 10 ng/ml of IL-3 and M-CSF, as described previously [61]. After incubation for 3 days (T3) /6 days (T6) they were collected for FACS sorting.

### Individual target validation

For paired guide RNA pDECKO-mCherry plasmid cloning we used the method described in [12] (sgRNA sequences are listed in Supplementary Table S1 and the cloning oligos are detailed in Supplementary Table S5). For single guide RNA pDECKO-mCherry plasmid cloning we used the method described in [62] (see Supplementary Table S6 for details of the oligos used). Plasmids constructed for this study can be found in Supplementary Table S7 (plasmids available at Addgene.org are indicated).

For lentivirus production, we co-transfected HeK293T virus packaging cells with 3 μg of each pDECKO_mCherry plasmid and packaging plasmids as described previously. Viral supernatant was collected 48 h post transfection and filtered through a cellulose acetate syringe filter. Polybrene (10 μg/ml) was added. We pelleted 5 × 10E5 BLaER1-Cas9 cells in two microcentrifuge tubes and resuspended each of them with 1 ml of viral supernatant. We performed spin-infection for 3 h at 1,000 g. After infection, the viral supernatant was removed and infected cells were resuspended with RPMI media supplemented with 10% heat-inactivated fetal bovine serum (FBS), 2 mM L-glutamine, and 100 U/ml Penicillin–Streptomycin. After 48 h of infection, we performed double selection with blasticidin (20 μg/ml) and puromycin (2 μg/ml) antibiotics. The selection was maintained for a minimum of 2 weeks.

BLaER1-Cas9 infected cells with the different pDECKO_mCherry plasmids were induced for transdifferentiation into macrophages at a density of 375,000 cells/mL by using 100 nM β-estradiol and 10 ng/ml of IL-3 and M-CSF, as described previously [61]. After incubation for 3 days (T3) /6 days (T6) the cells were analyzed by flow cytometry.

### Flow cytometry

For cell sorting: 30 × 10E6 cells were counted and resuspended in 300 μl PBS + 3% FBS in the presence of FcR blocking reagent. Cells were incubated for 10 min and 15 μl of the human anti-CD19 antibody conjugated with BV510 (Becton Dickinson, 562947) and 15 μl of human anti-cd11b (Mac1) antibody conjugated with PE-Cy7 (eBioscience, 25-0118-41) were added. Cells were incubated for 30 min in the dark, washed with PBS and resuspended in 2 ml of PBS + 3% FBS. Topro-3 was added as a viability marker. Cells were sorted in a BD FACS Aria instrument at the Flow Cytometry Unit of the Centre for Genomic Regulation.

For flow cytometry analysis: 1 × 10E6 cells were counted and resuspended in 100 μl PBS + 3% FBS in the presence of FcR blocking reagent. Cells were incubated for 10 min and 5 μl of each of the corresponding antibodies were added. For the CD19 knockout experiment, we used the antibody anti-CD19 conjugated with APC-Cy7 (Becton Dickinson, 557791). Cells were incubated for 30 min in the dark, washed with PBS and resuspended in 500 ul of PBS + 3% FBS. Topro-3 was added as a viability marker. Cells were measured in a BD Fortessa analyser. For the Stain Index calculation we used the formula: (mean positive—mean background) / (2 * SD background), as previously described [63].

Cell cytometry data is available in FlowRespository database (https://flowrepository.org) [64]. 

### Sample processing for deep sequencing

As a quality control, the pooled library was PCR amplified in two PCR steps, for the first PCR step it was used 50 ng of library for amplification with Phusion polymerase (Thermo Fisher) using oligos Stag0nt_F and Stag0nt_R (Supplementary Table S8), annealing temperature of 60ºC and 8 cycles of amplification. For the second PCR it was used 2 μl of purified PCR product from the previous step, amplified with the same conditions using an Illumina oligo pair (Supplementary Table S9). The final product was purified with Agencourt Ampure beads (Beckman Coulter), quantified with a Qubit fluorometer (Thermo Scientific), checked for quality in a Bioanalyzer (Agilent), and sequenced on the Illumina HiSeq 2500 at the Genomics Unit of the Centre for Genomic Regulation (125 bp paired-end sequencing).

After library infection, the genomic DNA was extracted from the FACS sorted cells with the GeneJET Genomic DNA purification kit (Thermo Scientific) and  two PCR steps were performed (see Fig. 3C). A scheme of oligo binding sites is shown in Supplementary Fig. S4.

A first PCR step was done by Phusion polymerase (Thermo Fisher) using 500 ng of genomic DNA and staggered oligo mix (Supplementary Table S8) with the presence of 6% DMSO, annealing temperature of 60ºC and a total of 20 cycles of amplification. We used staggered oligos to avoid the same bases being read for the constant region during Illumina sequencing and to minimize technical issues during base calling. Up to 6 PCR reactions were combined, the amplicons were gel-purified, and 2 ng were used as a template for a second PCR.

The second PCR step was also done by Phusion polymerase but without the presence of DMSO. We used Illumina barcoded oligos (Supplementary Table S9), an annealing temperature of 60ºC and a total of 8 cycles of amplification. Samples were purified with Agencourt Ampure beads (Beckman Coulter), quantified with a Qubit fluorometer (Thermo Scientific) and checked for quality in a Bioanalyzer (Agilent). We then pooled the libraries and sequenced them on the Illumina HiSeq 2500 at the Genomics Unit of the Centre for Genomic Regulation (150 bp paired-end sequencing) to have about 20 million reads per sorted subfraction. Sequencing data is available in the ArrayExpress database (http://www.ebi.ac.uk/arrayexpress) [65] under accession number E-MTAB-10445.

### Mapping and quantification of sequencing reads

For read mapping, based on the initial pgRNA library with two guides per target (Supplementary Table S2), an artificial genome was generated by concatenating the 41 bp of the two pgRNAs (gRNA1 21 bp, gRNA2 20 bp) and converted into FASTA format. STAR mapper (version 2.4.2a) [54] was used to index the genome, adjusting the standard settings by the following parameter for small genomes:

–genomeSAindexNbases 6.

In the resulting genome after removing duplicated constructs, each pgRNA pair is represented by each one of the 11,550 chromosomes with a length of 41 bp.

Dynamic trimming of Illumina reads was done in perl by pattern matching the insertion site of the pgRNAs in the plasmid sequence (“ACCG” for pgRNA1 in the window of 15–55 bp of read2, “AAAC” for pgRNA2 in the window of 100–150 bp of read1). The extracted 20 bp fastq sequences for the pgRNA2 were reverse-complemented and concatenated to the 21 bp fastq sequences for the pgRNA1. Fusion reads with fewer than 20 bp sequence length were filtered out.

Mapping was performed with STAR version 2.4.2a with the following parameters:

STAR –runMode alignReads –runThreadN 8 –genomeDir /users/resources/genome –readFilesCommand zcat –readFilesIn pgRNA1_pgRNA2.fastq.gz –alignIntronMax 1 –outSAMtype BAM SortedByCoordinate –outSAMunmapped Within –limitBAMsortRAM 3,000,000,000 –outFilterMultimapNmax 1 –outFilterMismatchNmax 11 –outFilterMatchNmin 30 –outFilterMatchNminOverLread 0.1 –outFilterMismatchNoverLmax 0.9 –outFilterScoreMinOverLread 0.1

Given the distance between the sequencing primer and gRNA2, the pipeline was conceived to be adjustable to a variable number of mismatches. Running the pipeline without allowing for any mismatches, we could only make use of about 25 to 30% of the reads. Hence, we increased the number of allowed mismatches in progressive steps that resulted in a steep increase of mapped reads until a saturation point was reached between 10–15 mismatches, depending on the sample (Supplementary Fig. S6C). For further analysis, we allowed for a maximum of 13 mismatches to stay below 1% of multi-mapped reads for all samples of both replicates. Spearman correlation values of 0.95–1.00 between samples, mapped with zero mismatches compared with up to 13 mismatches, justified the usage of the quantification data with substantially more reads and therefore higher statistical power (Supplementary Fig. S6D). For quantification, the count for each guide pair within the mapped libraries was aggregated from the BAM files with SAMtools [66].

Due to the low memory footprint of the artificial genome, this quantification strategy can be applied even on laptops with moderate specifications (minimum requirements: single core CPU, 4 GB RAM, 10 GB disk space). The mapped reads were clustered to check for reproducibility between replicates (data not shown).

### Analysis of the read counts

The count tables generated from the BAM files were filtered for guide pairs having at least 5 counts in the initial sample at T0, to ensure a minimum representation at the beginning of the experiment. For both biological replicates, the ratio of the FACS sorted delayed over differentiated fraction was computed for both T3 and T6. From the distribution of ratios from each of the 12,000 guide pairs, all guide designs found above the 90th percentile were selected. We further selected guide pairs for which both biological replicates of each time point had at least 2 guide designs for a given target above these 90th percentile in both time points separately.

### LNA GapmeRs assay

LNA antisense oligonucleotide GapmeRs (Exiqon) complementary to human lncRNA *LINC02432* (ENSG00000248810.1) (GCATGAAAGAGTTGGT) and lncRNA *MIR3945HG* (ENSG00000251230.1) (CTGAGAGGTGGCAAGC) were designed. A LNA oligonucleotide containing a scrambled sequence (AACACGTCTATACGC) was used as a negative control. We seeded 40,000 BLaER1 cells in a 24-well plate and the cells were grown in 1 ml complete RPMI media containing LNA GapmeRs at a final concentration between 1 and 2 μM. After 3 days of incubation, we induced transdifferentiation as described previously [61]. Total RNA was isolated from cells after 3 days of induction.

### RNA extraction, retro-transcription and quantitative PCR

RNA extractions from 1 × 10E6 cells were performed with Quick RNA Miniprep Kit (Zymo Research). 140 ng-500 ng RNA were retro-transcribed with Reverse Aid reverse transcriptase (Thermo Scientific). Quantitative PCR (qPCR) was performed with NZY Speedy qPCR Green Master mix (NZY tech) and in a LightCycler 480 Real-Time PCR System (Roche). Primer sequences are detailed in the Supplementary Table S10. Quantifications were normalized to an endogenous control (Glyceraldehyde 3-phosphate dehydrogenase, GAPDH). The relative quantification value for each target gene compared with the calibrator is expressed as 2^(-ΔΔCt).

### Western blot

1 × 10E6 cells were resuspended with 100 μL of Lysis buffer (1% SDS, 10 mM EDTA, 50 mM Tris pH 8, protease inhibitors). The cell lysate was sonicated in a Branson sonicator for 10 s (50% amplitude and power 7)*.* Protein concentration was checked by Pierce BCA protein assay kit (Thermo Fisher). The samples were run in a 10% SDS-PAGE gel and transferred to a nitrocellulose membrane. The membrane was blocked with blocking buffer (TBS, 0.1% Tween 20, 5% non fat milk) O/N at 4ºC, and incubated for 1 h 30’ at room temperature with primary antibodies: anti-FURIN rabbit polyclonal antibody (Proteintech, 18413–1-AP) 1:1,000 in blocking buffer, anti-NFE2 rabbit polyclonal antibody (Proteintech, 11089–1-AP) 1:1,000 in blocking buffer, or anti-CEBPa rabbit polyclonal antibody (Santa Cruz, (14AA): sc-61) 1:1,000 in blocking buffer. After 5 washes with TBS-0.1% Tween 20, the membranes were incubated for 1 h with the secondary antibody goat anti-rabbit-HRP (Sigma, G9545) 1:10,000 in blocking buffer. After 5 washes with TBS-0.1% Tween 20, the membranes were incubated either with Amersham ECL western blotting detection reagent (GE Healthcare, RPN2209), or Super Signal West Femto Maximum Sensitivity Substrate (Thermo Fisher, 34096), and imaged in an Amersham Imager 600. As a protein loading control, the membranes were re-blotted with primary antibody rabbit anti-GAPDH-HRP polyclonal antibody (Proteintech, 10494–1-AP) 1:4,000 in blocking buffer, and incubated for 1 h at room temperature. Washes and secondary antibody incubation were performed as previously described. The presence of two bands in NFE2 western blot likely corresponds to different post-translational modifications of NFE2 [28]. We used the following protein ladders: Supersignal molecular weight protein ladder (Life Technologies, 84785) and pre-stained Spectra multicolor broad range protein ladder (Life Technologies, 26634).

### TA cloning

In order to sequence the edited region in BLaER1-Cas9 cells, we amplified the deletion junctions by PCR using oligos outside the cut region (Supplementary Table S11). The resulting PCR products were cloned using a TA cloning kit (Invitrogen-Life Technologies) or Topo TA cloning kit (Invitrogen-Life Technologies), according to manufacturer’s instructions. We performed colony PCR and the purified product was sequenced by Sanger sequencing.

### Analysis of interacting regions by ABC

Data on significant enhancer-gene interactions was retrieved from the *Activation By Contact* study [37]. Interactions from the following available cell lines on the lymphoid and myeloid branches were subset from the total number of cell lines: B cells, GM12878, Karpas 422, BJAB, CD19-positive B cells, CD14-positive monocytes, u-937 and THP1 cells. Enhancers closer than 100 bp were merged. Correlations of expression across time between lncRNA and interacting pc-genes were computed on the average of the two replicates.

## Supplementary Information

**Additional file 1: Supplementary Figure S1.** Expression clusters of lncRNAs during transdifferentiation. **Supplementary Figure S2.** Expression clusters of protein coding genes during transdifferentiation. **Supplementary Figure S3.** Expression profiles of lncRNAs and protein coding genes during transdifferentiation. **Supplementary Figure S4.** pDECKO plasmid and sequencing oligos binding scheme. **Supplementary Figure S5.** FACS sorting of BLaER1-Cas9 library. **Supplementary Figure S6.** Statistics on quantification of pgRNA representation in the screening. **Supplementary Figure S7.** Quantification of pgRNA distribution before and during screening. **Supplementary Figure S8.** Target genes disrupted by CRISPR-Cas9. **Supplementary Figure S9. **CEBPa and SPI1 validation at genomic level. **Supplementary Figure S10.** Western blot of CEBPa. **Supplementary Figure S11.** Individual target validation by flow cytometry. **Supplementary Figure S12.** lncRNA target sites and individual validations. **Supplementary Figure S13.** Validation of lncRNAs knockout at genomic level. **Supplementary Figure S14**: Epigenetic landscape of the candidate lncRNAs**. Supplementary Figure S15.** FURIN and NFE2 target sites and validationsat genomic level. **Supplementary Figure S16.** Uncropped western blots.

**Additional file 2: Supplementary Table S1.** gRNA sequences. **Supplementary Table S3.** Shortlisted candidates from screening. **Supplementary Table S4.** RNA-Seq expression data of target genes during differentiation(in FPKMs). **Supplementary Table S5.** Oligos for pgRNA cloning in pDECKO_Cherry. **Supplementary Table S6.** Oligos for sgRNA cloning of CD19 in pDECKO_mCherry. **Supplementary Table S7.** List of cloned plasmids. **Supplementary Table S8. **Sequences of staggered Oligos **Supplementary Table S9.** Sequences of Illumina Oligos (barcode in brackets). **Supplementary Table S10.** Sequences of oligos for qRT-PCR. **Supplementary TableS11.** Oligos outside the CRISPR cut region.

**Additional file 3: Supplementary Table S2:** CRISPETa output lists for controls and targets including final CRISPR 165 oligonucleotide library.

## Data Availability

Cell cytometry data is available in FlowRespository database (https://flowrepository.org) [64] under accession links: https://flowrepository.org/id/FR-FCM-Z3PD, https://flowrepository.org/id/FR-FCM-Z3PG, https://flowrepository.org/id/FR-FCM-Z3PH, https://flowrepository.org/id/FR-FCM-Z3PJ, https://flowrepository.org/id/FR-FCM-Z3PK, https://flowrepository.org/id/FR-FCM-Z3PL, https://flowrepository.org/id/FR-FCM-Z3PM, https://flowrepository.org/id/FR-FCM-Z3PW. Sequencing data is available in the ArrayExpress database (http://www.ebi.ac.uk/arrayexpress) [65] under accession number E-MTAB-10445. BLaER1 pgRNA CRISPR library and individual pDECKO_mCherry plasmids are available at Addgene.org under the following accession numbers: library ID 183825, plasmids IDs 140098 to 140109.

## Abbreviations

ABC: Activation By Contact
Avg: Average
BCA: Bicinchoninic Acid
BFP: Blue Fluorescent Protein
CEACAM: Carcinoembryonic Antigen-Related Adhesion Molecule
CRISPR: Clustered Regularly Interspaced Short Palindromic Repeats
CRIPSRdel: CRISPR deletion
CRISPRi: CRISPR inhibition
CRISPR-ko: CRISPR knockout
dCas9: Nuclease-dead Cas9
DDE: Differentiation Delaying Effect
DECKO: Dual excision CRISPR knockout
EGFP: Enhanced Green Fluorescent Protein
ER: Estrogen Receptor
FACS: Fluorescence-Activated Cell Sorting
FBS: Fetal Bovine Serum
FPKM: Fragments Per Kilobase Of Exon Per Million Mapped Reads
GAPDH: Glyceraldehyde-3-Phosphate Dehydrogenase
H3K27ac: Histone 3 Lysine 27 acetylation
HRP: Horseradish Peroxidase
KRAB: Krüppel-Associated Box
IL-3: Interleukin 3
indel: Insertion/deletion
KLF4: Krüppel-like factor 4
lncRNA: Long non-coding RNA
M-CSF: Macrophage Colony-Stimulating Factor
NFE2: Nuclear Factor Erythroid 2
ORF: Open Reading Frame
PAM: Protospacer Adjacent Motif
PBS: Phosphate-Buffered Saline
pc: Protein coding
PCR: Polymerase Chain Reaction
pgRNA: Paired guide RNA
qPCR: Quantitative PCR
RNA-seq: RNA sequencing
RT-qPCR: Reverse Transcription qPCR
SD: Standard Deviation
sgRNA: Single guide RNA
TBS: Tris-Buffered Saline
TREML2: Trem-Like Transcript 2
TSS: Transcription Start Site
