# Supplementary material for: Paired guide RNA CRISPR-Cas9 screening for protein-coding genes and lncRNAs involved in transdifferentiation of human B-cells to macrophages
Source: BMC Genomics. 2022 May 26;23:402. doi: 10.1186/s12864-022-08612-7 (PMC9137126; doi:10.1186/s12864-022-08612-7)
Supplement: Supplementary file 1 — Additional file 1: Supplementary Figure S1. Expression clusters of lncRNAs during transdifferentiation. Supplementary Figure S2. Expression clusters of protein coding genes during transdifferentiation. Supplementary Figure S3. Expression profiles of lncRNAs and protein coding genes during transdifferentiation. Supplementary Figure S4. pDECKO plasmid and sequencing oligos binding scheme. Supplementary Figure S5. FACS sorting of BLaER1-Cas9 library. Supplementary Figure S6. Statistics on quantification of pgRNA representation in the screening. Supplementary Figure S7. Quantification of pgRNA distribution before and during screening. Supplementary Figure S8. Target genes disrupted by CRISPR-Cas9. Supplementary Figure S9. CEBPa and SPI1 validation at genomic level. Supplementary Figure S10. Western blot of CEBPa. Supplementary Figure S11. Individual target validation by flow cytometry. Supplementary Figure S12. lncRNA target sites and individual validations. Supplementary Figure S13. Validation of lncRNAs knockout at genomic level. Supplementary Figure S14: Epigenetic landscape of the candidate lncRNAs. Supplementary Figure S15. FURIN and NFE2 target sites and validationsat genomic level. Supplementary Figure S16. Uncropped western blots. [file 12864_2022_8612_MOESM1_ESM.pdf]

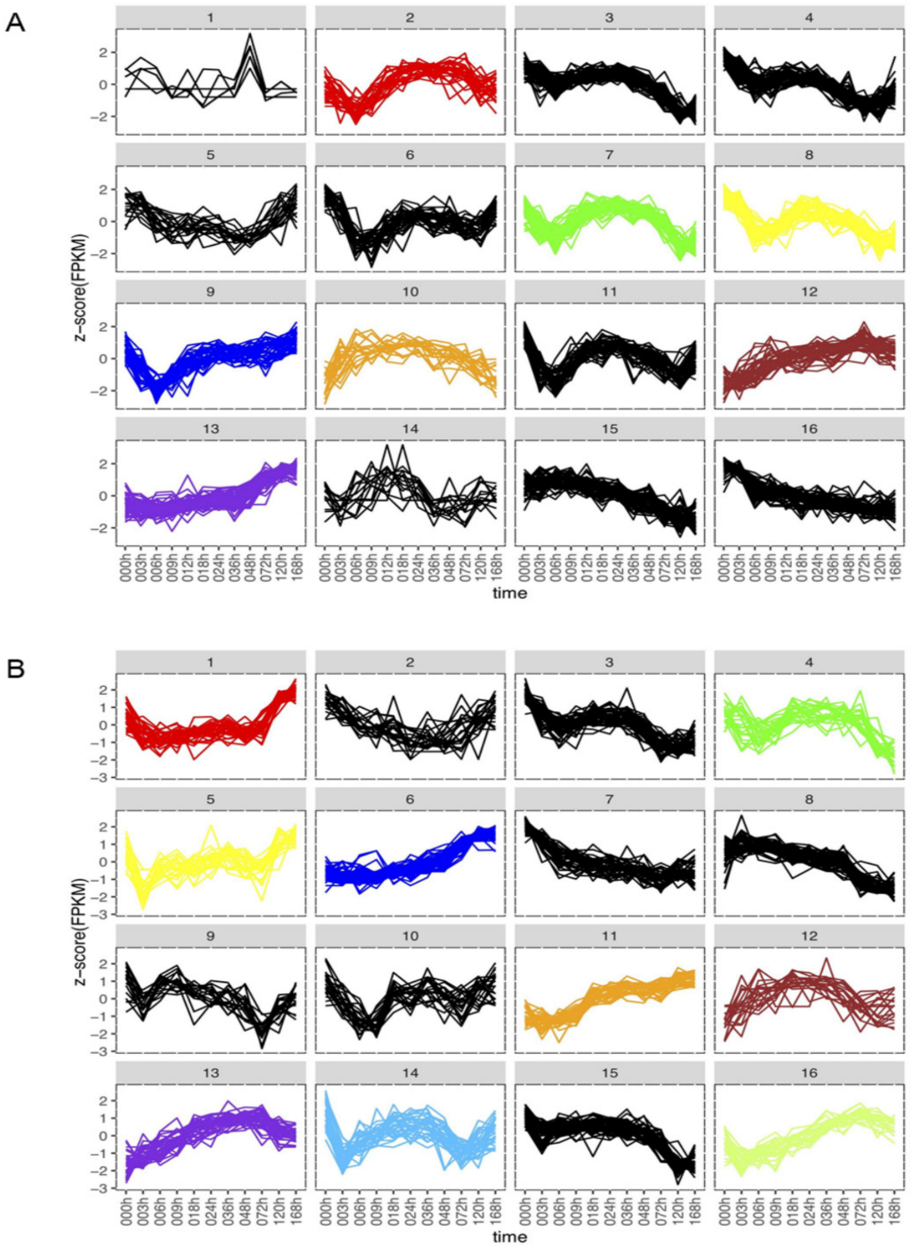

Supplementary Figure S1: Expression clusters of lncRNAs during transdifferentiation.

Clusters of lncRNAs with similar expression characteristics during transdifferentiation of BLaER1 cells to macrophages. lncRNAs clustered into 16 expression profiles by k-means clustering. FPKM values were log10 transformed and normalized by z-transformation. Results from 2 biological replicates are shown (**A**) and (**B**). Clusters used for the CRISPR library are highlighted using the colour that labels the genes in Figure 1C.

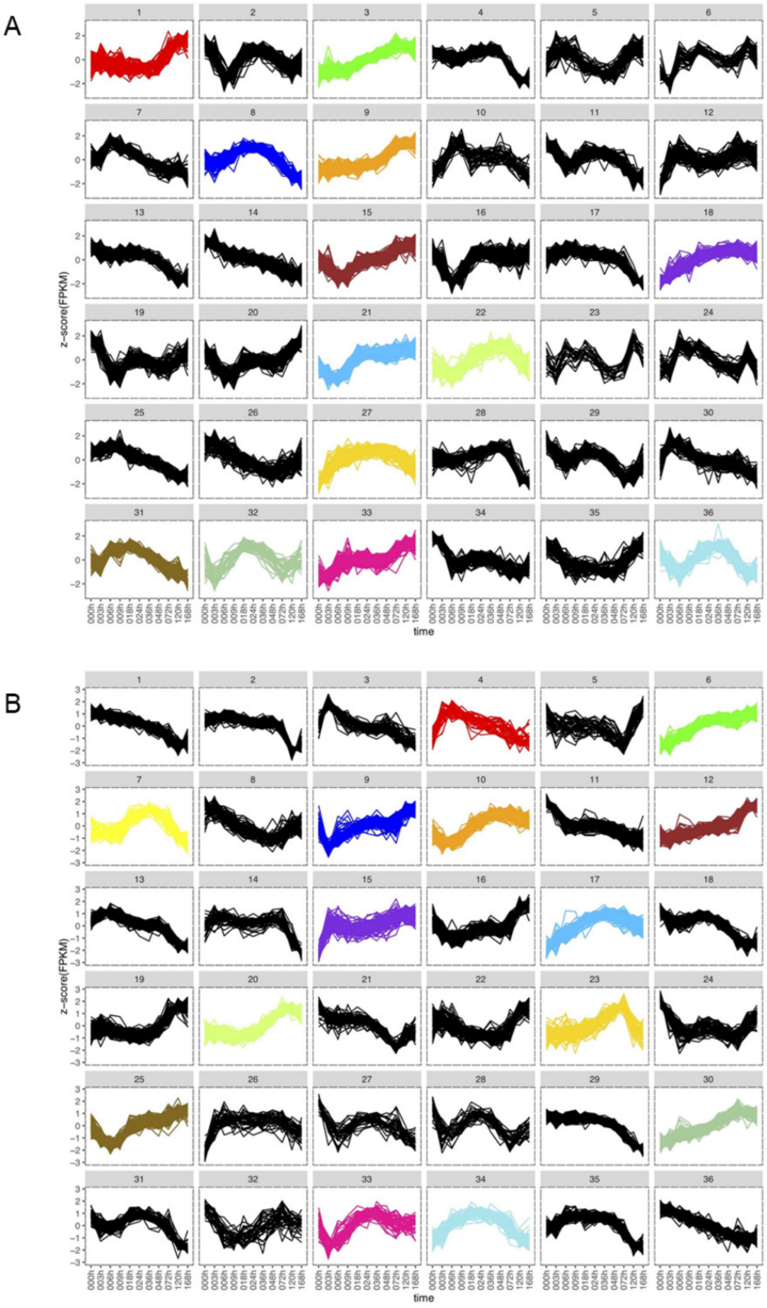

Supplementary Figure S2: Expression clusters of protein coding genes during transdifferentiation. Clusters of protein coding genes with similar expression characteristics during transdifferentiation of BLaER1 cells to macrophages. Pc-genes clustered into 36 expression profiles by k-means clustering. FPKM values were log10 transformed and normalized by z-transformation. Results from 2 biological replicates are shown (**A**) and (**B**). Clusters used for the CRISPR library are highlighted using the color that labels the genes in Figure 1C.

A

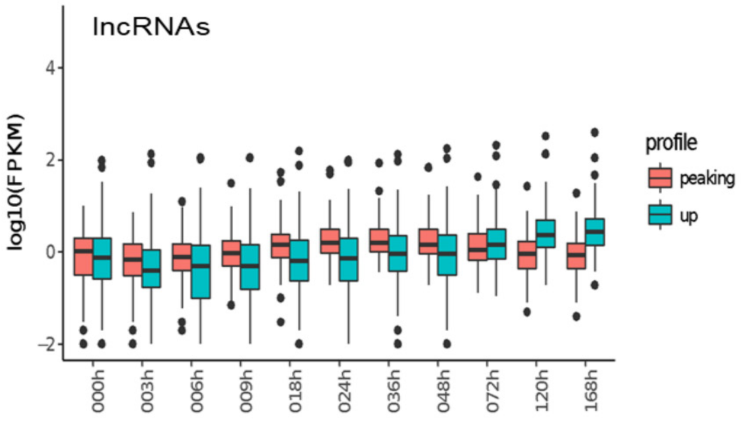

B

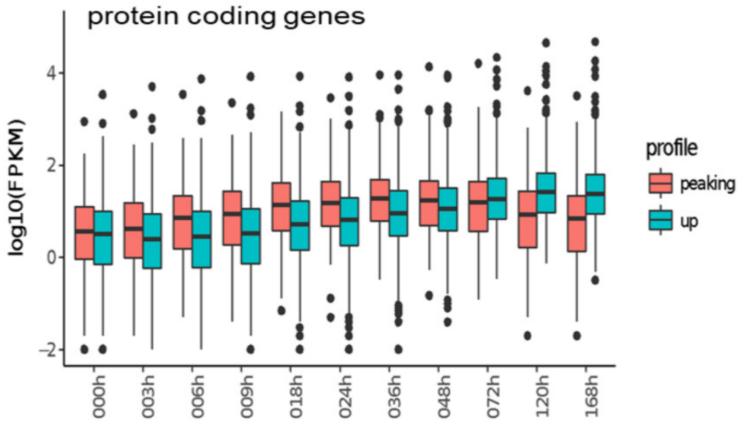

Supplementary Figure S3: **Expression profiles of lncRNAs and protein coding genes during transdifferentiation.**

Log10 transformed expression profiles of the 163 lncRNAs (**A**), and of the 939 protein coding genes (**B**), with peaking (red) or increasing (blue) expression.

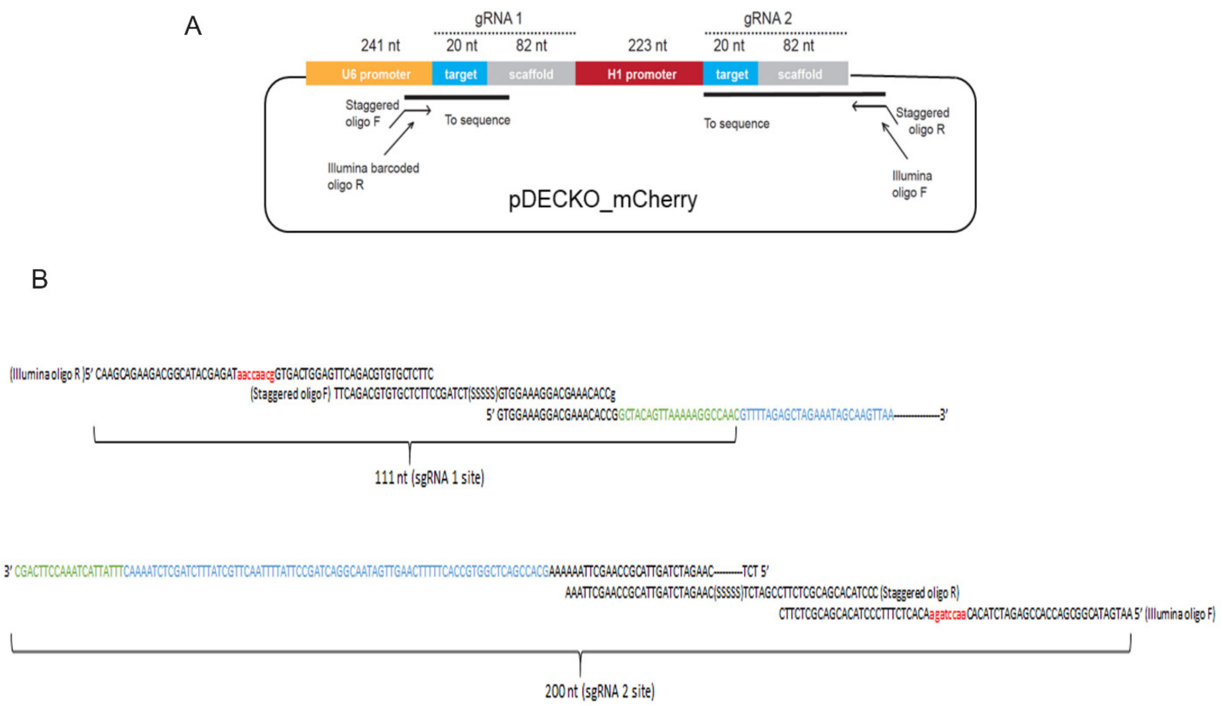

Supplementary Figure S4: **pDECKO plasmid and sequencing oligos binding scheme.**

(**A**) Scheme of pDECKO_mCherry plasmid. H1 and U6 promoters drive the expression of the two gRNAs. The primers of the two PCR amplification steps performed before deep sequencing are also shown (see also Supplementary Tables S8 and S9). In the first PCR step, staggered oligos anneal to the U6 promoter and to the pDECKO backbone. In the second PCR step, primers containing the sample barcode and P5/P7 sequences for sequencing are added. (**B**) Oligo binding sites are shown for Illumina sequencing amplicons. The amplicon length (until the sgRNA) is 111 nt for the forward strand and 200 nt for the reverse strand. The constant scaffold sequences and the variable sgRNAs are shown in blue and green respectively. 1st PCR oligos (Staggered oligos) are shown with the staggered oligos labeled with S. 2nd PCR oligos (Illumina oligos) are shown with the barcode sequences indicated in red (see also Supplementary Tables S8 and S9).

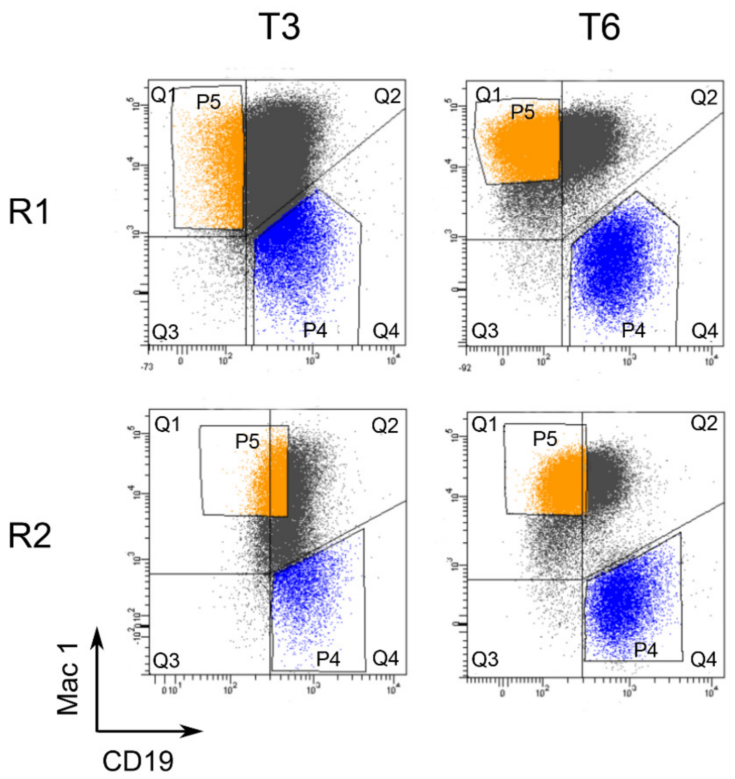

		Q1	Q2	Q3	Q4	P4	P5
R1	T3	10.6%	72.5%	0.8%	16%	11.7%	8.9%
	T6	46.1%	39.7%	0.5%	13.8%	13.4%	38.5%
R2	T3	12.4%	73.8%	1.3%	12.5%	10.7%	31.3%
	T6	46.8%	37.2%	0.7%	15.3%	14.7%	49%

Supplementary Figure S5: FACS sorting of BLaER1-Cas9 library.

BLaER1-Cas9 cells infected with the pDECKO CRISPR library were transdifferentiated for T3 and T6. The cells were stained with antibodies against surface markers CD19 for B-cells and Mac1 for macrophages, and FACS sorted. Sorted gates for cells delayed in transdifferentiation (gate P4 in blue) and cells normally differentiating (gate P5 in orange) are shown for two biological replicates (R1 and R2). The percentage of cells in each quadrant and gates is indicated.

A

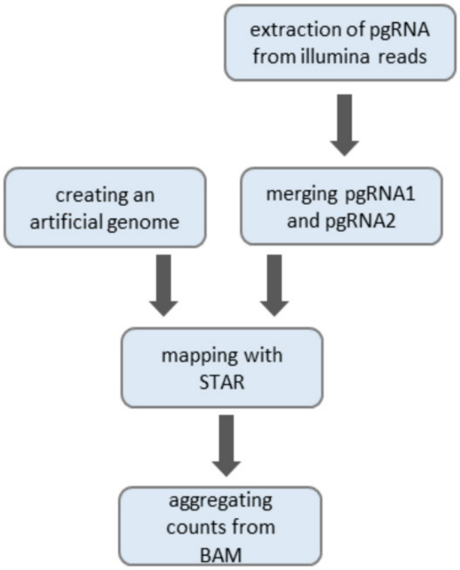

B

	T0_total	T3_total	T6_total	T3_diff	T3_del	T6_diff	T6_del
**Total reads**	23174328	20461721	17622205	33261946	35163035	21620659	28360044
**Uniquely mapped reads**	13648684	12123869	10320734	18582918	19804799	12840942	15755565
**Uniquely mapped reads %**	58.90%	59.25%	58.57%	55.87%	56.32%	59.39%	55.56%
**Ambiguous reads**	120157	106017	102014	165894	195573	107748	235855
**Ambiguous reads %**	0.52%	0.52%	0.58%	0.50%	0.56%	0.50%	0.83%
**Unmapped reads**	40.52%	40.15%	40.78%	43.55%	43.06%	40.03%	43.56%

	T0_total	T3_total	T6_total	T3_diff	T3_del	T6_diff	T6_del
**Total reads**	26644511	26966759	25395692	24415795	33691107	20150115	24517712
**Uniquely mapped reads**	17547956	17617769	16316787	15763071	18564557	12993696	14626243
**Uniquely mapped reads %**	65.86%	65.33%	64.25%	64.56%	55.10%	64.48%	59.66%
**Ambiguous reads**	131929	138169	138888	116321	216878	99229	169568
**Ambiguous reads %**	0.50%	0.51%	0.55%	0.48%	0.64%	0.49%	0.69%
**Unmapped reads**	33.55%	34.06%	35.10%	34.82%	44.15%	34.90%	39.55%

C

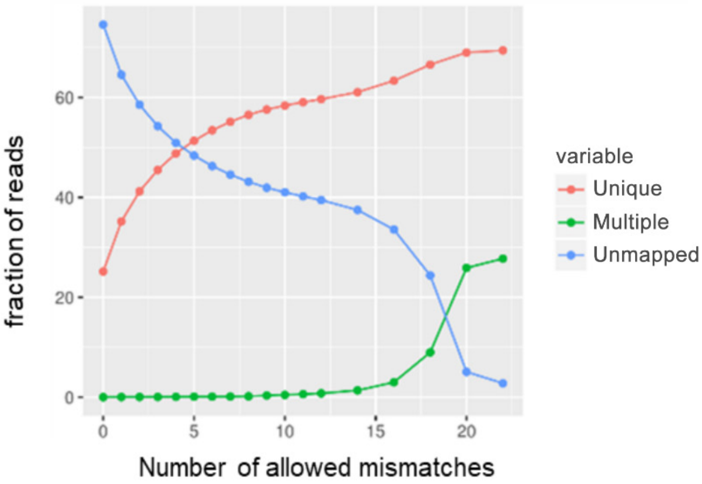

D

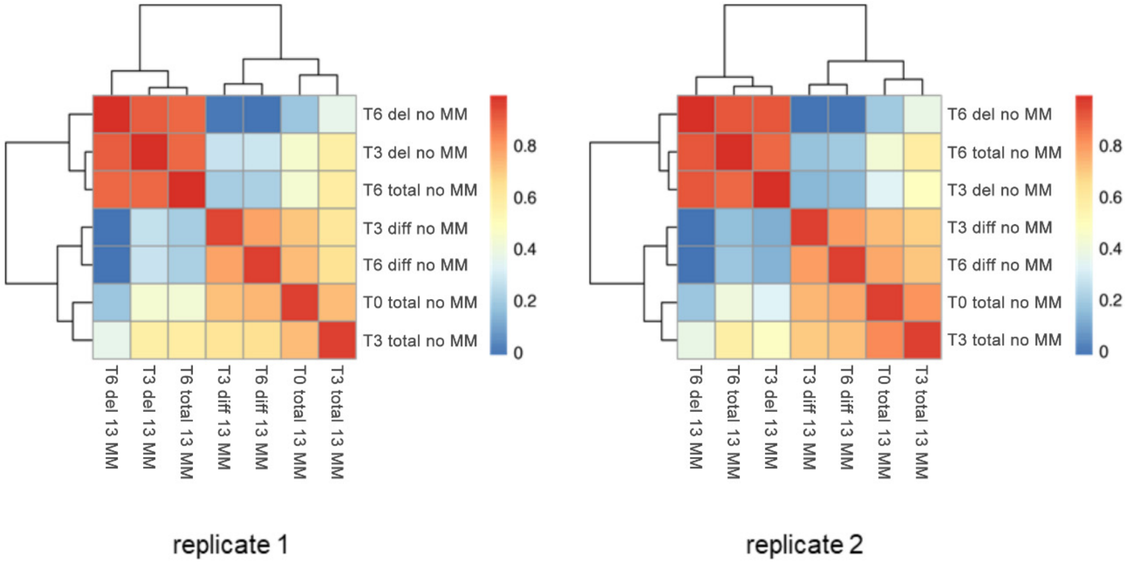

Supplementary Figure S6: Statistics on quantification of pgRNA representation in the screening.

(**A**) Schematic flow diagram displaying the steps from the pgRNA FASTQ sequences after sequencing to count tables per pgRNA. In short, pgRNA sequences are extracted from FASTQ sequences by finding the proximal constant plasmid sequence. pgRNA2 is reverse complemented (only needed for paired end sequencing) and merged with pgRNA1. Both are mapped as one sequence to the merged expected sequences converted into artificial chromosomes with STAR mapper. Count tables are generated from BAM files by aggregation. (**B**) Detailed mapping statistics for both replicates in the quantification. Initial read counts per sample ranged between 20 to 35 million reads of which on average 55% can be mapped against perfect library sequences with not more than 13 mismatches (diff = differentiated population, del = population with delayed differentiation). The high proportion of unmapped reads stems from the position of one of the two gRNAs at the end of the 150 bp fragment. Decreased sequencing quality at the end of the read impacts the proportion of total mapped reads. (**C**) Uniquely mapped, multi-mapped and unmapped reads as a function of allowed mismatches during quantification of the sequenced samples in a range of 0 to 26 mismatches. (**D**) Pearson correlations of guide pair quantification between runs allowing for up to 13 mismatches and only allowing perfect matches. Correlation values for identical source samples were ranging between 0.95 and 1. (diff = differentiated cell population from FACS orange gate P5, del = cell population with delayed differentiation from FACS blue gate P4). Results for two biological replicates are shown.

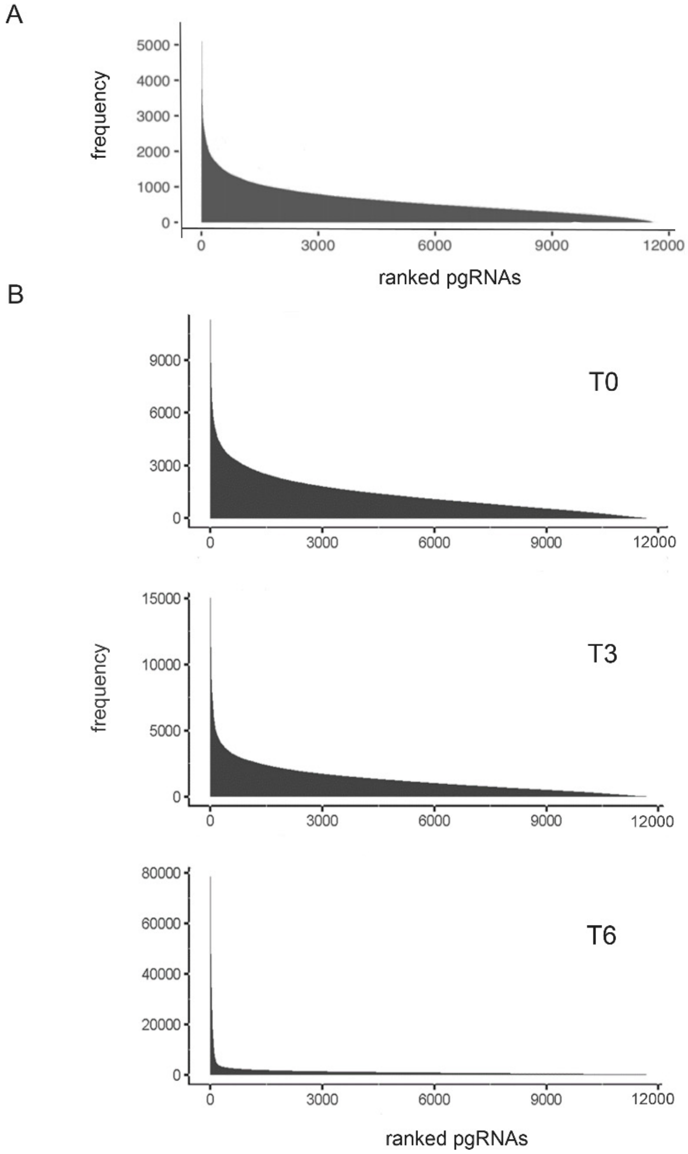

Supplementary Figure S7: Quantification of pgRNA distribution before and during screening.

(**A**) Ranked distribution of counts per pgRNA in the initial library (after cloning). The library showed a good representation and homogeneity (> 95 % of coverage and a delta of top to bottom decile < 10 fold, respectively). (**B**) Ranked distribution of counts per pgRNA in the control samples at T0, T3 and T6 upon transdifferentiation induction, that contain all cells independent of B-cell and macrophage marker abundance.

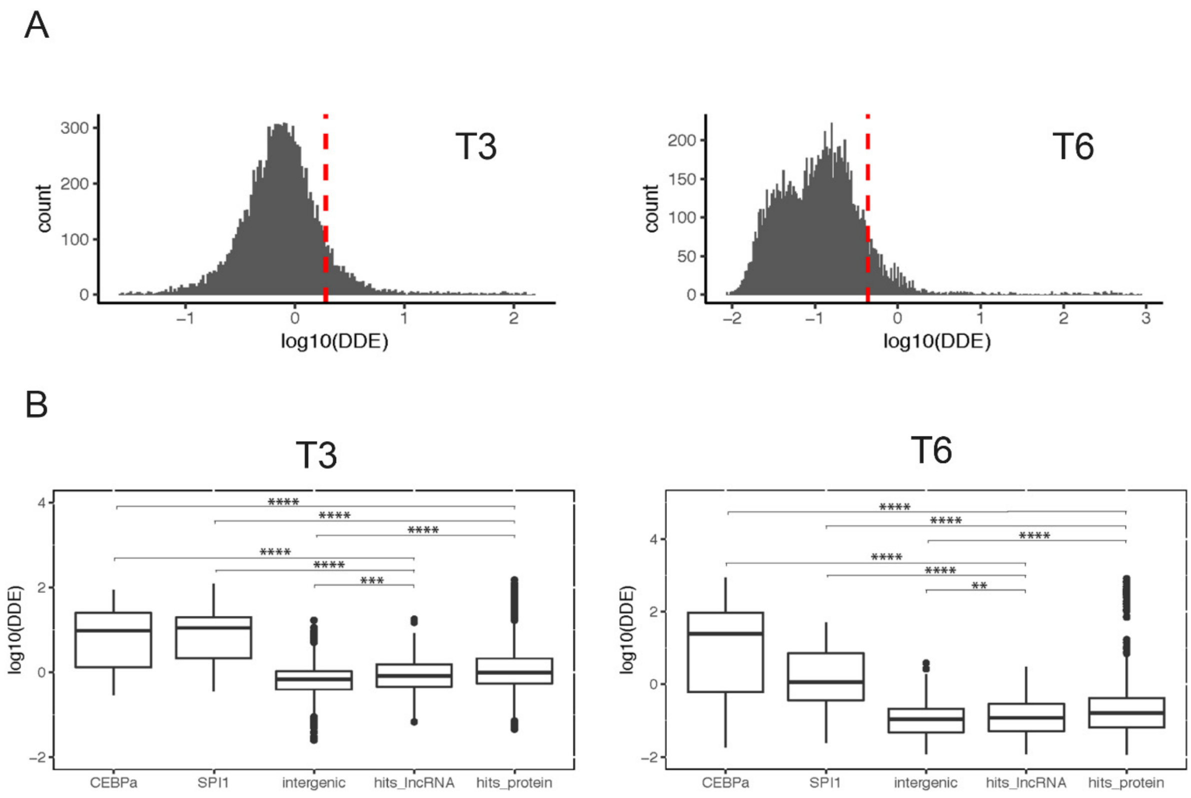

Supplementary Figure S8: Target genes disrupted by CRISPR-Cas9.

(**A**) Distribution of DDE values at T3 and T6 upon transdifferentiation induction. DDE is computed as the ratio of normalized counts from the delayed subpopulation (del) divided by the counts from the transdifferentiated population (dif). Highest decile is marked by a dashed red line. Values are the average between the two biological replicates. (**B**) Comparison of DDE values for the selected candidate pgRNAs targeting lncRNAs and protein coding genes of the highest decile (from panel A) at T3 and T6 upon transdifferentiation induction, with *CEBPa* and *SPI1* as positive controls, and intergenic regions as negative controls. Values are the average between the two biological replicates. Significant values were assessed by means of the Wilcoxon test.

A

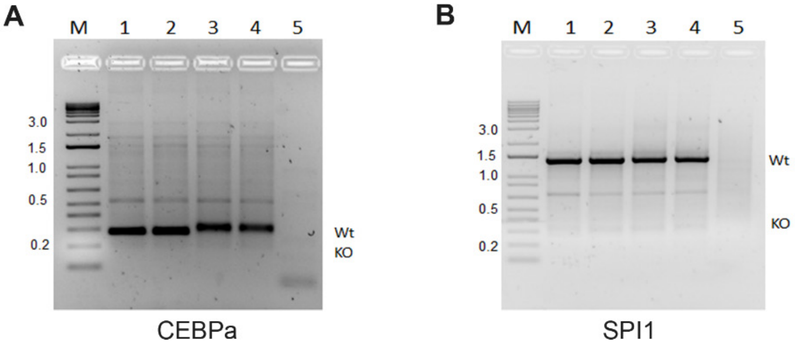

C

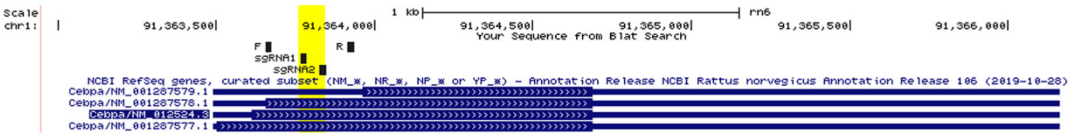

D

Wt AGGTCGGCCAGGAACTCGTCGTTGAAGGCGGCCGGGTCGATGTAGGCGCTGATGTCTATAGACGTCTCGTGCTCGCAGATGCCGCCCAGCGGCTCCGGGGCGGCA

Cut AGGTCGGCCAGGAACTCGTCGTTGA/--/CAGCGGCTCCGGGGCGGCA

Cl1 AGGTCGGCCAGGAACTCGTCGTT/----/GGCGGCCGGGTCGATGTAGGCGCTGATGTCTATAGACGTCTCGTGCTCGCAGATG/--------/CCTATTCCCCGGGGCGGCA

Cl2 AGGCAGGAACTCGTCGTTGAAAGAGGGAAGGCCGGGTCGATGTAGGCGCTGATGTCTATAGACGTCTCGTGCTCG/------------------/CAGGAGCTCCGGGGCGGCA

Cl3 AGGTCGGCCAGGAACTCGTCGTTGAGCTCCCCGGGTCGATGTAGGCGCTGATGTCTATAGACGTCTCGTGCTCGCAGATGCCGC(I10)CAGCGGCTCCGGGGCGGCA

Cl4 AGGTCGGCCAGGAACTCGT-GTTGAAGGCGGCCGGGTCGATATAGGCGCTGATGTCTATAGACGTCTCGT/--/GCTCCGGGGCGGCA

E

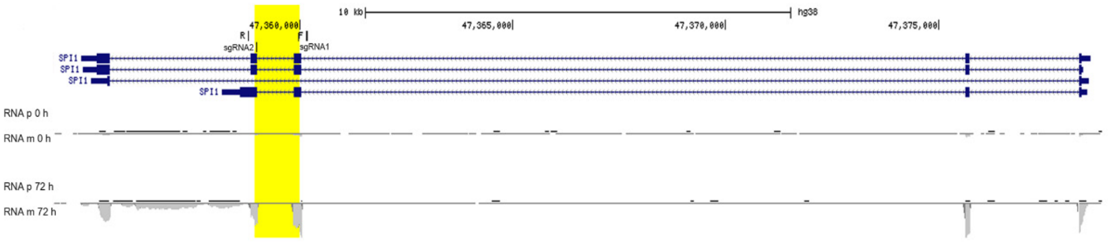

F

Wt GGATGGGTACTGGAGGCACATCCGGGGCAGGTA(.............................991............................)ACTCGCTGTGCACGTGGTGGGGGTGG

Cut GGATGGGTACTGGAGGCACA/--/TGGGGGTGG

Cl 1 GGATGGGTACTGGAGGCACATTCTCCTCCGGGGCAGGTA(...................991......................)ACTCGCTGTGCACGTGGGCTGGGGGTGG

Cl 2 GGATGGGTACTGGAGGCACACAGGAACCTCCGGGGCAGGTA(................991..............)ACTCGCTGTGCACGTGGGCCCTGGGGGTGG

Cl 3 GGATGGGTACTGGAGGCACAAGACTCCGGGGCAGGTA(.................991.................)ACTCGCTGTGCACGTGGGTCTTTCTGGGGGTGG

Cl 4 GGATGGGTACTGGAGGCACAGGTCCGGGGCAGGTA(.......................991.....................)ACTCGCTGTGCACGTGGGGGCTGGGGGTGG

Cl 5 GGATGGGTACTGGAGGCACAGTTCCTCCGGGGCAGGTA(........................991....................)ACTCGCTGTGCACGTGGCTTGGGGGTGG

Supplementary Figure S9: CEBPa and SPI1 validation at genomic level.

(**A** and **B**) PCR products from genomic DNA of: (1) BLaER control cells, (2) pDECKO-Intergenic BLaER-Cas9 control cells, (3 and 4) pDECKO-CEBPa CRISPR-Cas9 edited cells (in **A**) or pDECKO-SPI1 CRISPR-Cas9 edited cells (in **B**) from two biological replicates, (5) water control. (M) 1 Kb molecular weight ladder. Expected molecular weights are (**A**) for *CEBPa* 278 nt for the wild type band (Wt) and 217 nt for the knockout band (KO) and (**B**) for *SPI1* 1,384 nt for the wild type band and 356 nt for the knockout band. (**C**) UCSC genome browser diagrams of *ratCEBPa* locus. (**D**) Sanger sequencing of *ratCEBPa* locus from clones of pDECKO-CEBPa infected cells (from TA cloning). (**E**) UCSC genome browser diagrams of human *SPI1* locus. (**F**) Sanger sequencing of *SPI1* locus from clones of pDECKO-SPI1 infected cells (from TA cloning). In (**C**) and (**E**), pgRNAs binding sites are highlighted in yellow. Primers used for checking CRISPR deletion are indicated as F/R. PAM sequences are shown in green, insertions are shown in red and deletions are shown as dashed lines. For *SPI1* (**F**) the presence of the original gene sequence (991 bp) between the edited target sites is also indicated.

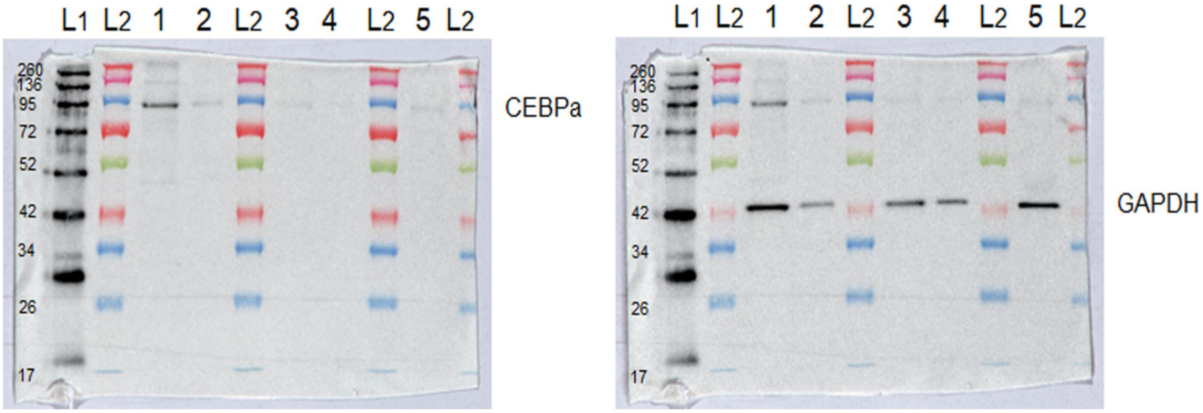

Supplementary Figure S10: **Western blot of CEBPa.**

Western blots corresponding to CEBPa (left) and GAPDH (right) protein expression in BLaER-Cas9 cells: (1) BLaER cells, positive control for CEBPa expression, (2) pDECKO-Intergenic at T0, (3) pDECKO-CEBPa at T0 replicate 1, (4) pDECKO-CEBPa at T0 replicate 2, and (5) pDECKO-CEBPa at T0 replicate 2 loading double amount of protein. (L1) Supersignal western blot protein ladder (150 kDa, 100 kDa, 80 kDa, 60 kDa, 50 kDa, 40 kDa, 30 kDa, 20 kDa) and (L2) multicolor protein ladder (260 kDa, 135 kDa, 95 kDa, 72 kDa, 52 kDa, 42 kDa, 34 kDa, 26 kDa, 17 kDa). Expected protein molecular weights are: 73.6 kDa for CEBPa fused to Estrogen Receptor [26] and 36 kDa for GAPDH.

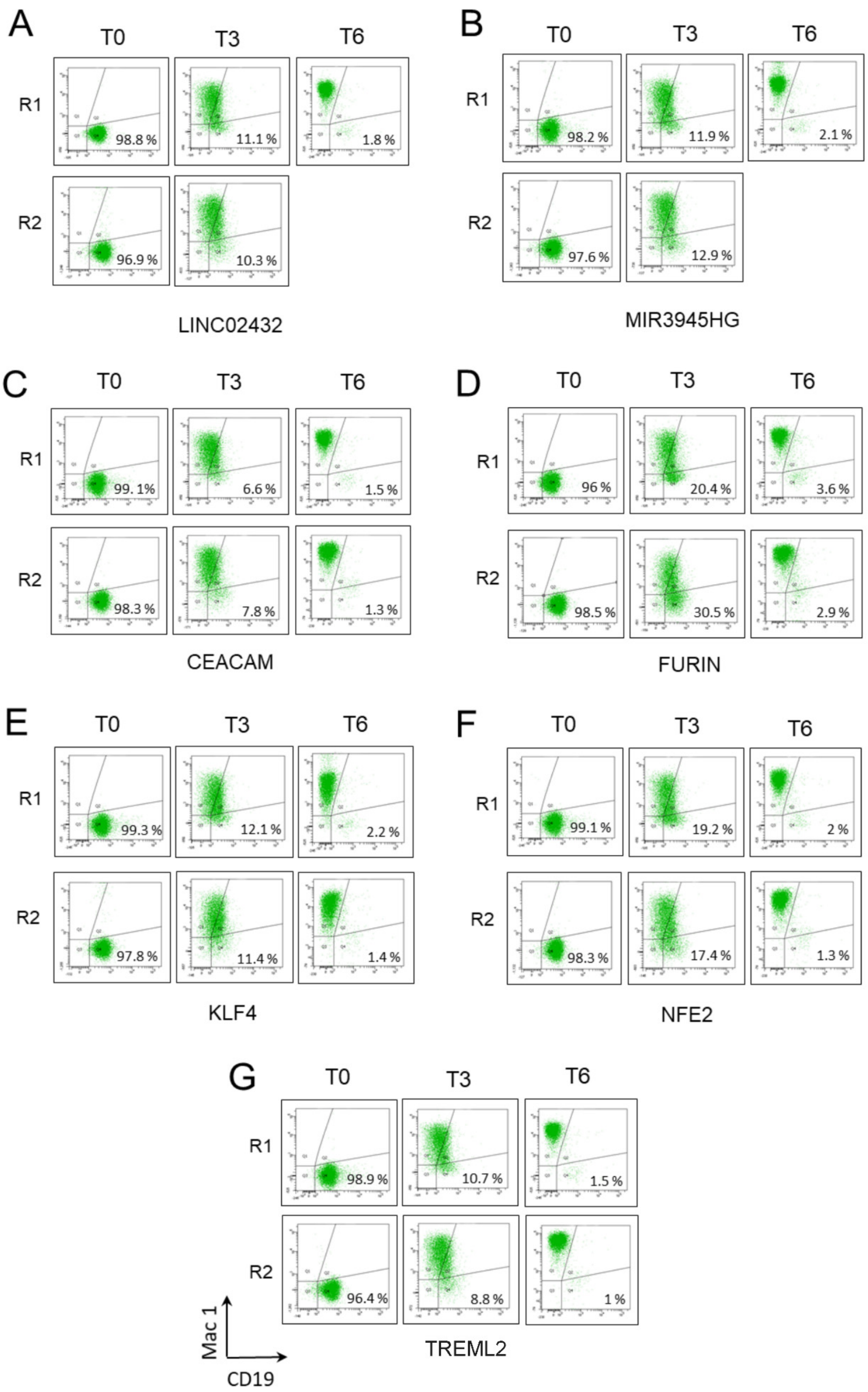
A
T0
T3
T6
R1
98.8 %
11.1 %
1.8 %
R2
96.9 %
10.3 %
LINC02432
B
T0
T3
T6
R1
98.2 %
11.9 %
2.1 %
R2
97.6 %
12.9 %
MIR3945HG
C
T0
T3
T6
R1
99. 1%
6.6 %
1.5 %
R2
98.3 %
7.8 %
1.3 %
CEACAM
D
T0
T3
T6
R1
96 %
20.4 %
3.6 %
R2
98.5 %
30.5 %
2.9 %
FURIN
E
T0
T3
T6
R1
99.3 %
12.1 %
2.2 %
R2
97.8 %
11.4 %
1.4 %
KLF4
F
T0
T3
T6
R1
99.1 %
19.2 %
2 %
R2
98.3 %
17.4 %
1.3 %
NFE2
G
T0
T3
T6
R1
98.9 %
10.7 %
1.5 %
R2
96.4 %
8.8 %
1 %
Mac 1
CD19
TREML2

Supplementary Figure S11: Individual target validation by flow cytometry.

Flow cytometry analysis of targets validated from the screening at 0, 3, and 6 days (T0, T3 and T6) of transdifferentiation. CD19 was used as a B-cell marker on the X-axis and Mac1 was used as a macrophage marker on the Y-axis. Cells that do not undergo transdifferentiation (delayed fraction) remain in quadrant Q4 (percentages of cells for this quadrant are shown at T3 and T6 upon transdifferentiation induction). (**A**) *LINC02432*; (**B**) *MIR3945HG*; (**C**) *CEACAM*; (**D**) *FURIN*; (**E**) *KLF4*; (**F**) *NFE2*; (**G**) *TREML2*. Two biological replicates (R1 and R2) are shown except for *LINC02432* and *MIR3945HG* from which only one biological replicate was available at T6.

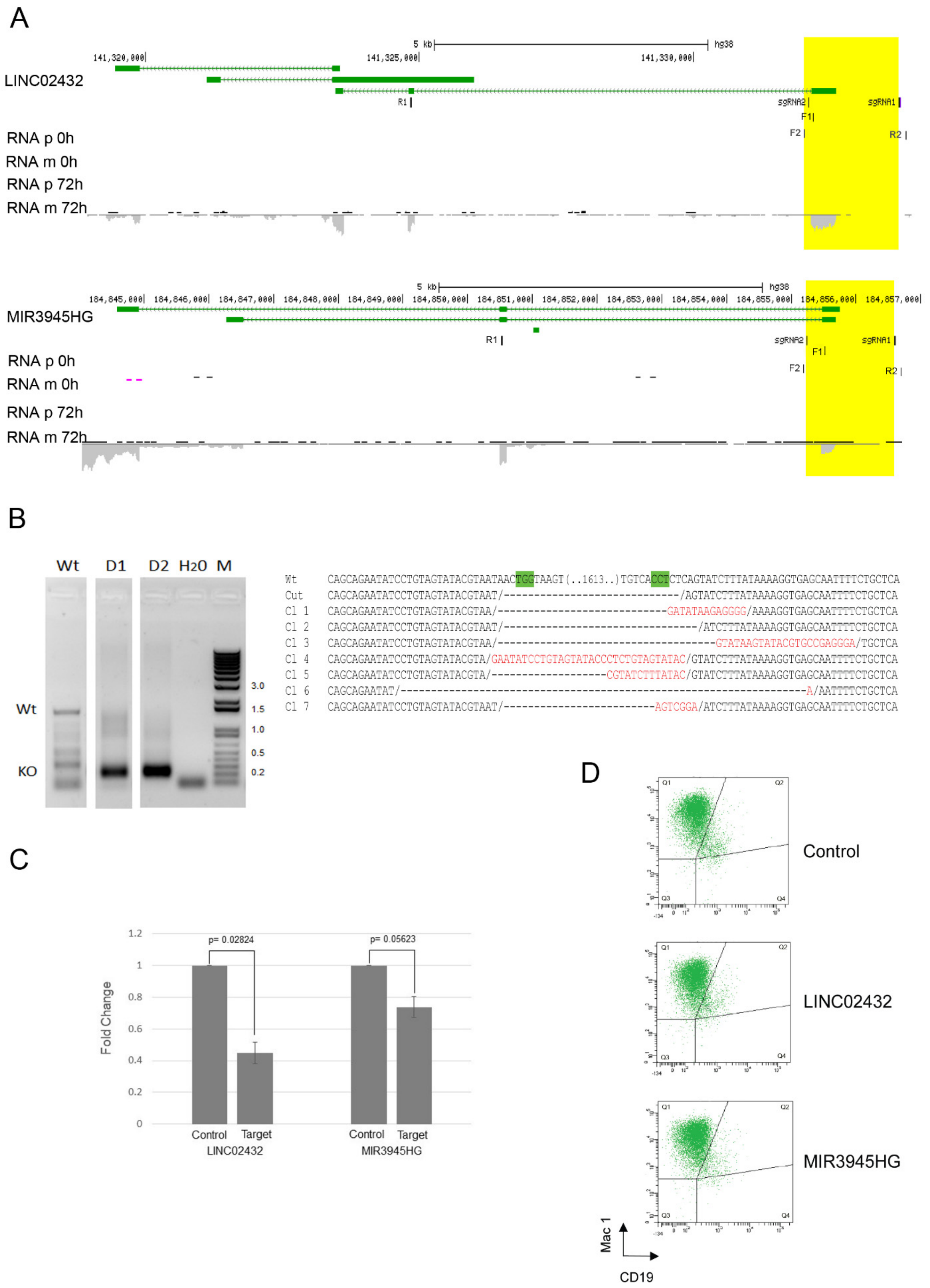
A
5 kb
hg38
141,320,000
141,325,000
141,330,000
LINC02432
R1
sgRNA2
sgRNA1
F1
F2
R2
RNA p 0h
RNA m 0h
RNA p 72h
RNA m 72h
184,845,000
184,846,000
184,847,000
184,848,000
184,849,000
184,850,000
184,851,000
184,852,000
184,853,000
184,854,000
184,855,000
184,856,000
184,857,000
MIR3945HG
B
Wt
D1
D2
H2O
M
Wt
KO
3.0
1.5
1.0
0.5
0.2
Wt CAGCAGAATATCCTGTAGTATACGTAATAACTGGTAAGT(..1613..)TGTCACCTCTCAGTATCTTTATAAAAGGTGAGCAATTTTCTGCTCA
Cut CAGCAGAATATCCTGTAGTATACGTAAT/------------------------------/AGTATCTTTATAAAAGGTGAGCAATTTTCTGCTCA
Cl 1 CAGCAGAATATCCTGTAGTATACGTAA/-----------------------------GATATAAGAGGGG/AAAAGGTGAGCAATTTTCTGCTCA
Cl 2 CAGCAGAATATCCTGTAGTATACGTAAT/---------------------------------/ATCTTTATAAAAGGTGAGCAATTTTCTGCTCA
Cl 3 CAGCAGAATATCCTGTAGTATACGTAA/-------------------------------------GTATAAGTATACGTGCCGAGGGA/TGCTCA
Cl 4 CAGCAGAATATCCTGTAGTATACGTA/GAATATCCTGTAGTATACCCTCTGTAGTATAC/GTATCTTTATAAAAGGTGAGCAATTTTCTGCTCA
Cl 5 CAGCAGAATATCCTGTAGTATACGTA/--------------------CGTATCTTTATAC/GTATCTTTATAAAAGGTGAGCAATTTTCTGCTCA
Cl 6 CAGCAGAATAT/--A/AATTTTCTGCTCA
Cl 7 CAGCAGAATATCCTGTAGTATACGTAAT/--------------------------AGTCGGA/ATCTTTATAAAAGGTGAGCAATTTTCTGCTCA
C
Fold Change
1.2
1
0.8
0.6
0.4
0.2
0
p= 0.02824
p= 0.05623
Control
Target
LINC02432
Control
Target
MIR3945HG
D
Control
LINC02432
MIR3945HG
Q1
Q2
Q3
Q4
Mac 1
CD19

Supplementary Figure S12: lncRNA target sites and individual validations.

(A) Diagrams from UCSC genome browser showing the binding sites of pgRNAs (in yellow) for *LINC02432* and *MIR3945HG*. qPCR primers binding sites are indicated as F1/R1 and primers for checking CRISPR cut are indicated as F2/R2. RNA seq expression is shown at 0h and 72h of transdifferentiation. (**B**) Left, agarose gel showing the PCR products from genomic DNA amplification of wild type cells (wt) or *LINC02432* CRISPR-Cas9 edited cells (delayed fraction FACS sorted enriched cell population) for two biological replicates (D1 and D2), a water template (H20) was used as a PCR negative control, (M) 1 Kb molecular weight ladder. The gel was cropped for more clarity, see the uncropped gel in Supplementary Fig. S13A. Wild type and knockout bands have an expected size of 1,817 nt and 179 nt respectively. Right, Sanger sequencing results from clones obtained with TA cloning method (PAM sequences are shown in green, insertions are shown in red and deletions are shown as dashed lines). (**C**) qRT-PCR to check the expression of the lncRNAs *LINC02432* and *MIR3945HG* after GapmeR treatment and cell differentiation for 3 days (T3) in BLaER1 cells treated with GapmeR control, or BLaER1 cells treated either with GapmeRs against *LINC024332* or *MIR3945HG* (Target). The values correspond to the average of 2 biological replicates, one-tail t-test p-values are indicated. (**D**) Flow cytometry analysis at T3 of differentiation of BLaER cells treated with GapmeR control (against an intergenic region), or GapmeRs against *LINC02432* and *MIR3945HG*.

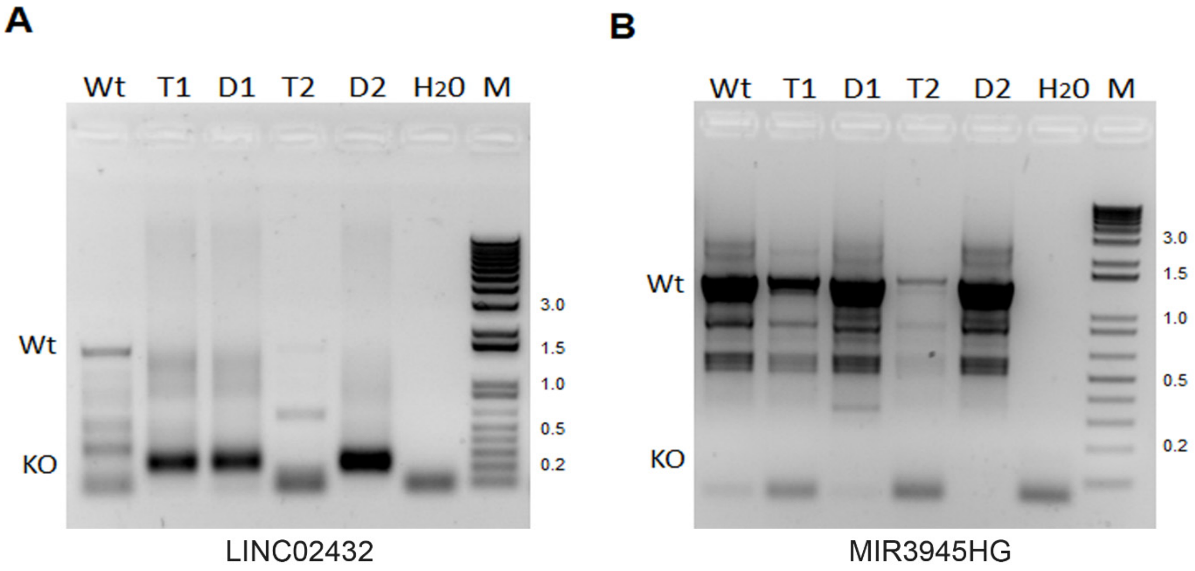

Supplementary Fig. S13: **Validation of lncRNAs knockout at genomic level.**

Agarose gels showing the PCR products from pDECKO-LINC02432 (**A**) or pDECKO-MIR3945HG (**B**) BLaER-Cas9 edited cells (from FACS sorted enriched populations) for two biological replicates. Gel A is the uncropped agarose gel corresponding to Supplementary Fig. S12B. (Wt) pDECKO-Intergenic BLaER-Cas9 control cells, (T1 and T2) transdifferentiated population sorted cells of two biological replicates, (D1 and D2) delayed population sorted cells for two biological replicates, (H_20) water control, (M) 1 Kb molecular weight ladder. Expected bands are 1,817 bp for negative control (Wt) and 179 bp for KO of pDECKO-LINC02432 (**A**), and 1,504 bp for negative control (Wt) and 128 bp for KO of pDECKO-MIR3945HG (**B**). For *MIR3945HG*, although no bands with the expected size after the induced deletion are observed, some bands different from the wild type ones appear in lanes D1 and D2, suggesting some specific genomic rearrangement after CRISPR-Cas9 deletion (**B**).

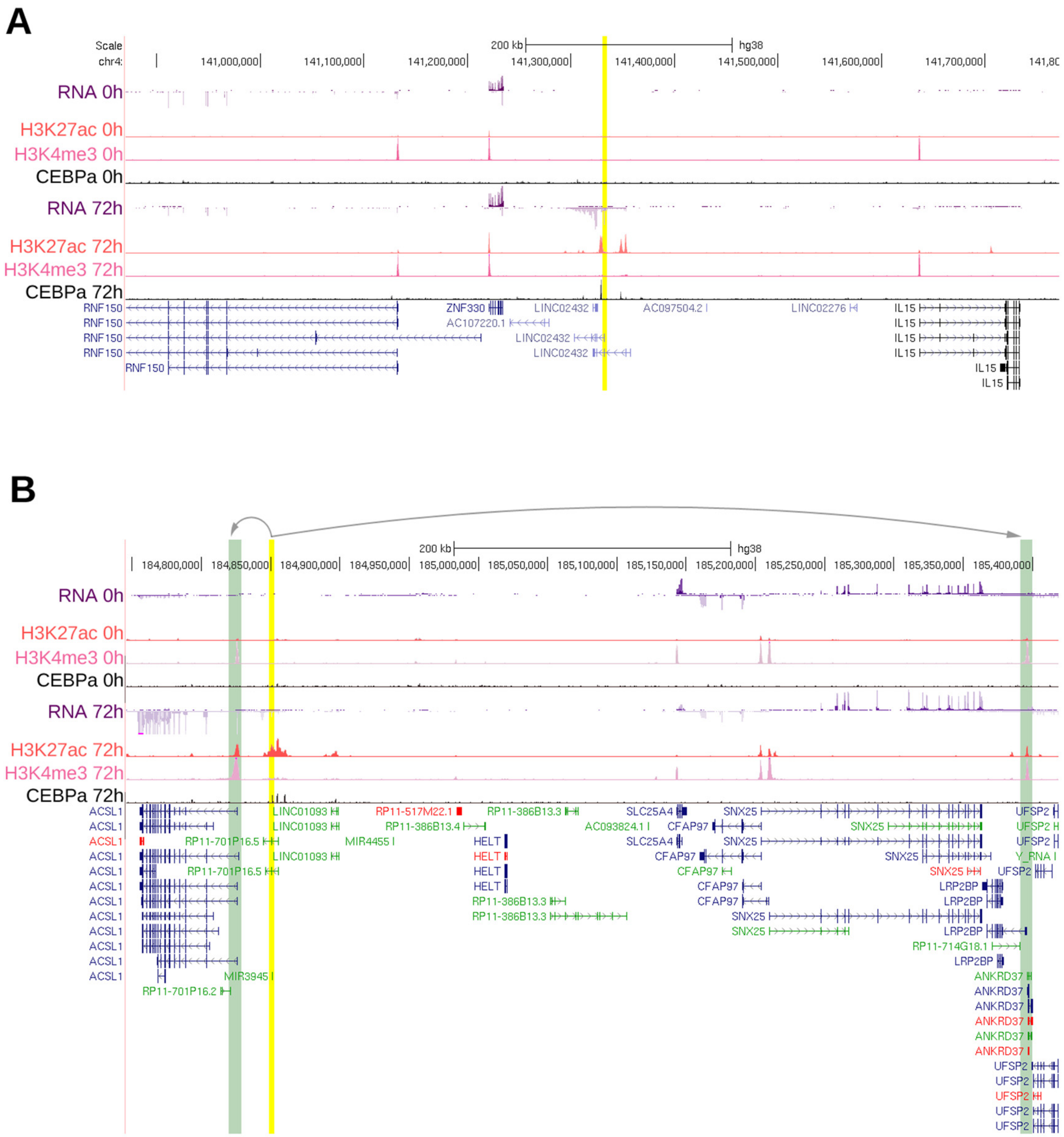

Supplementary Figure S14: Epigenetic landscape of the candidate lncRNAs.

UCSC genome browser diagrams for *LINC02432* (**A**) and *MIR3945HG* (**B**). Regions deleted by specific pgRNAs are highlighted in yellow. In both cases, there is an increase of H3K27ac mark [27], associated with active promoters and enhancers, overlapping or close to the deleted region and coinciding with the binding of CEBPa [36] and the upregulation of the lncRNAs expression at 72h after induction. There is no increase in H3K4me3 mark, typically associated with active promoters. This is consistent with these regions behaving as enhancers. In **B**, genes contacting the deleted region around *MIR3945HG* TSS are highlighted in green and contacts are depicted as gray arrows.

A

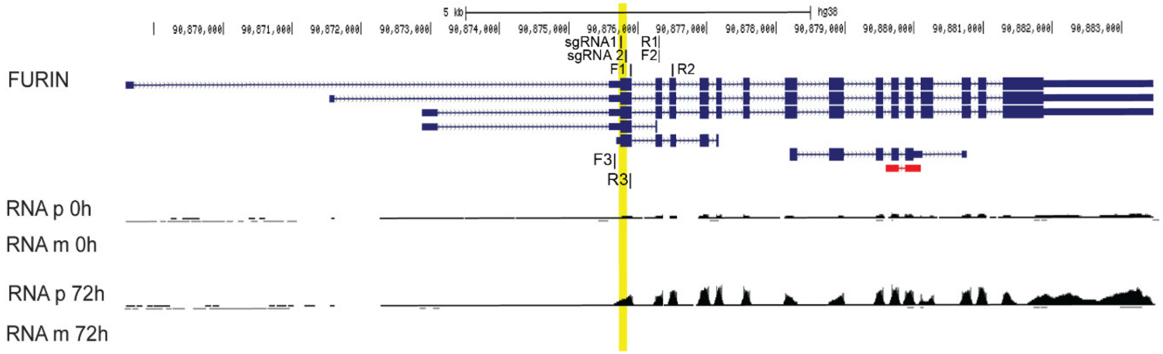

B

Wt CCCTGGTTGCTATGGGTGGTAGCAGCAACAGGAACCTTGGTCCTGCTAGCAGCTGATGCTCAGGGCCAGAAGGTCTTCACCAACACGTGGGCTGTGCGCAT

KO CCCTGGTTGCTAT/ --/ACGTGGGCTGTGCGCAT

cl 1 CCCTGGTTGCTAT/ ------------------------------ TTGCTATAGGACCCC ------------------------------/ACGTGGGCTGTGCGCAT

cl 2 CCCTGGTTGCTAT/ ------------------------------ TTGCTAT- GG ------------------------------/ACGTGGGCTGTGCGCAT

cl 3 CCCTGGTTGCTAATGGGTGGTAGCAGCAACAGGAACCTTAGTCCTGGTCCTGCTAGCAGCTGATGCTCAGGGCCAGAAGGTCTTCACCAACCGGACGTGGGCTGTGCGCAT

cl 4 CCCTGGTTGCTAGGGCTGGGTGGTAGCAGCAACAGGAACCTTGGTCCTGCTAGCAGCTGATGCTCAGGGCCGGAAGGTCTTCACCAACCCACGTGGGCTGTGCGCAT

cl 5 CCCTGGTTGCTAGAGACGGCGCCGTCGTGCTCCCCGGAGTGGAGGCGGCCGAGCGCGCCGGGGTGCCCGCCTTCCTGGAGACCTCCGCGCCCCGCAACCTCCCCTTCTACGAGGCTGTGCGCAT

cl 6 CTGNTTGCC/------------------------------AACCTTGGTCCTGCTAGCAGCTGATGCTCAGGGCCAGAAGGTCTTCACCAAC----GTGGGCTGTGCGCAT

cl 7 CCCTGGTTGCTATGCCGGGGTGGTAGCAGCAACAGGAACCTTGGTCCTGCTAGCAGCTGATGCTCAGGGCCAGAAGGTCTTCACCAAGGGCCGTGGGCTGTGCGCAT

C

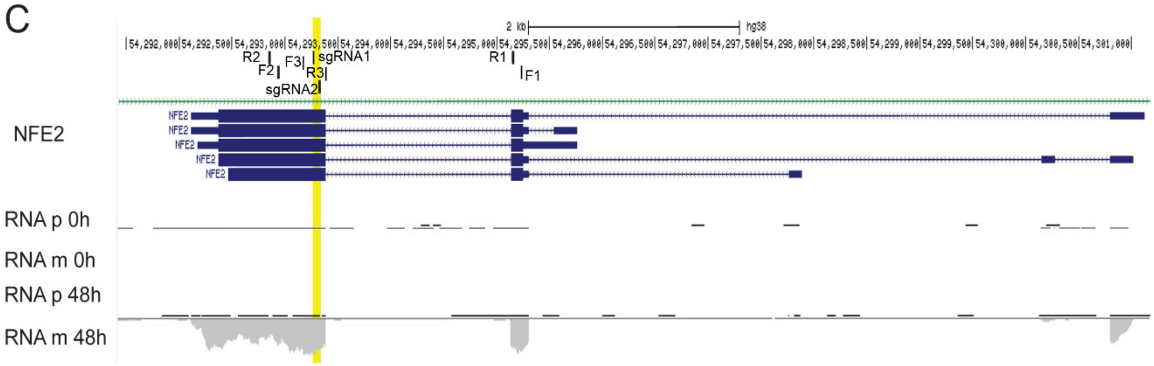

D

Wt AGGAAGTGGGAAGCCAGAATCTGGGTGGATTGAGCAGGGGCAGTAAGTTGTGGGTGGTGGAGGTCCAAGGTATGGAGCTGGGGCTT

KO AGGAAGTGGGAAGCCAGAA/--/AGCTGGGGCTT

cl 1 AGGAAGTGGGAAGC/----------/CTGGGTGGATTGAGCAGGGGCAGTAAGTTGTGGGTGGTGGAGGTCCAAGGTATGGCAGCNTGGCNG

cl 2 AGGAAGTGGGAAGCCAGAATCTGGGTGGATTGAGCAGGGGCAGTAAGTTGTGGGCGGTGGA---NTCCAAGC/--/TG-/--/GCTG

cl 3 AGGAAGTGGGAAGCCAGAAAGACTCTGGGTGGATTGAGCAGGGGCAGTAAGTTGTGGGTGGTGGAGGTCCAAGGTA/-----/AGCTG

cl 4 AGGAAGTGGGAAGCCAGAAACGAACACTCTCTGGGTGGATTGAGCAGGGGCAGTAAGTTGTGGGTGGTGGAGGTCCAAGGTA

Supplementary Figure S15: FURIN and NFE2 target sites and validations at genomic level.

UCSC genome browser diagrams for *FURIN* (**A**) and *NFE2* (**C**). pgRNAs binding sites are highlighted in yellow. The two primer pairs used for qRT-PCR are indicated as F1/R1 and F2/R2. Primers used for checking CRISPR deletion are indicated as F3/R3. Sanger sequencing results are shown from clones obtained with TA cloning: (**B**) for pDECKO-FURIN infected cells and (**D**) for pDECKO-NFE2 infected cells. PAM sequences are shown in green, insertions are shown in red and deletions are shown as dashed lines.

A

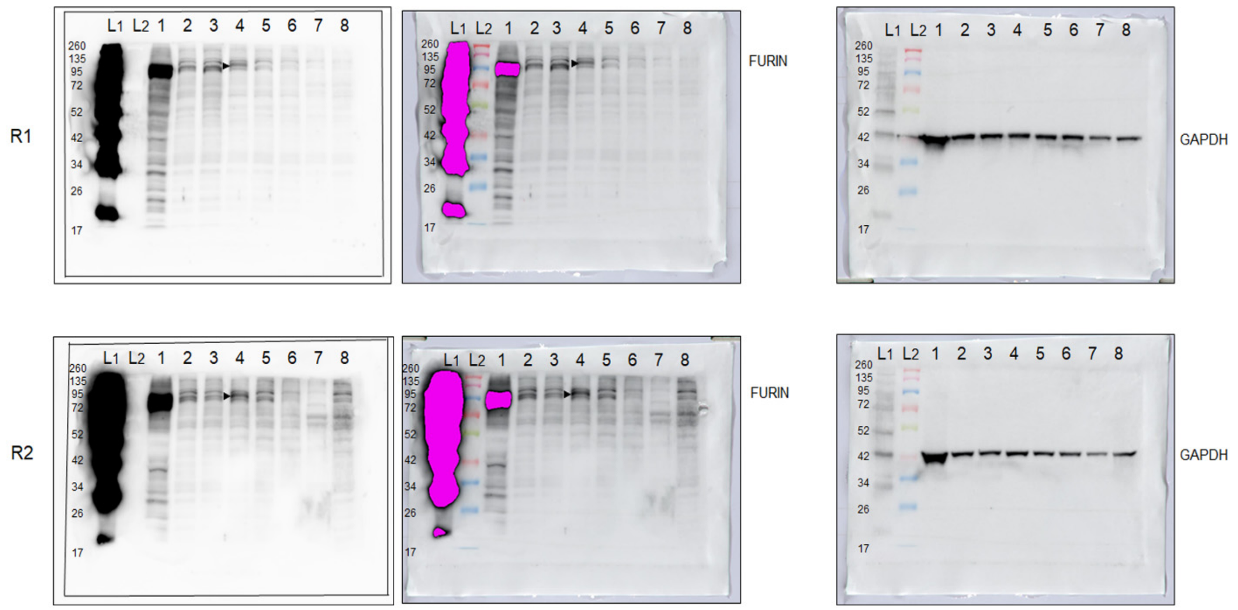
R1
R2
L1 L2 1 2 3 4 5 6 7 8
260
135
95
72
52
42
34
26
17
FURIN
GAPDH

B

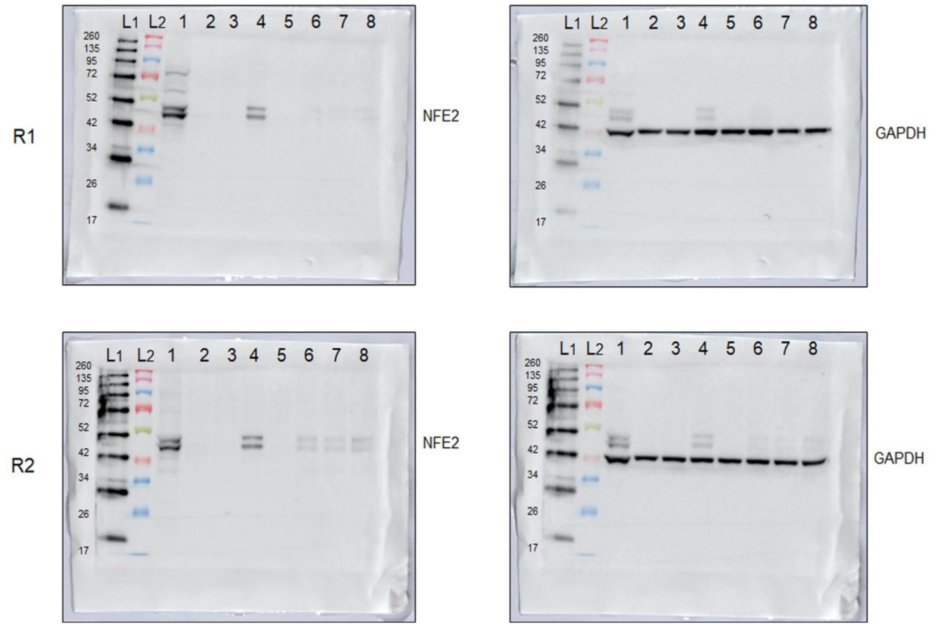
R1
R2
L1 L2 1 2 3 4 5 6 7 8
260
135
95
72
52
42
34
26
17
NFE2
GAPDH

Supplementary Figure S16: Uncropped western blots.

(A) Uncropped western blots corresponding to FURIN protein expression in BLaER-Cas9 (see Fig. 6A) for two biological replicates (R1 and R2). The Amersham Imager 600 (AI600) that we have used for the analysis of the western results, gave two different output images (on the left hand side, we show a blot without the multicolor pre-stained ladder from which we have indicated the membrane edges – the cropped image sown in Fig. 6A comes from this type of image shown in replicate two (lanes 3 to 7) -; on the right hand side, we show the same blot under exactly the same experimental conditions but with the pre-satined multicolor ladder automatically displayed, the AI600 machine highlighted in pink all the saturated bands – see lane L1 and lane 1 -, in this case the gel edges are clearly visible. Lanes are as follows: (L1) Supersignal molecular weight protein ladder, (L2) pre-stained Spectra multicolor broad range protein ladder, (1) HeLa cells, positive control for FURIN, (2) BLaER-Cas9 cells at T0, (3, CT0) and (4, CT3) control pDECKO-Intergenic at T0 and T3 respectively, (5, FUT0) and (6, FUT3) pDECKO-FURIN at T0 and T3, (7, FUT3s) pDECKO-FURIN at T3 and sorted from gate P4 (delayed population), (8) pDECKO-FURIN at T3 and sorted from gate P7 (transdifferentiated population). The same membranes were re-blotted with an antibody against GAPDH and are shown on the right hand side of the image. Exposition time was 1 minute for all the membranes. (**B**) Uncropped western blots corresponding to NFE2 protein expression (see Fig. 6B) for two biological replicates (R1) and (R2). The cropped image showed in Fig. 6B correspond to replicate 2 (lanes 3 to 7). The lanes are as follows: (L1) Supersignal molecular weight protein ladder, (L2) pre-stained Spectra multicolor broad range protein ladder, (1) K562 cells, positive control for NFE2, (2) BLaER-Cas9 cells at T0, (3, CT0) and (4, CT2) control pDECKO-Intergenic at T0 and T2 respectively, (5, NFT0) and (6, NFT2) pDECKO-NFE2 at T0 and T2, (7, NFT2s) pDECKO-NFE2 at T2 and sorted from gate P4 (delayed population), (8) pDECKO-NFE2 at T2 and sorted from gate P7 (transdifferentiated population). The same membranes were re-blotted with an antibody against GAPDH and are shown on the right hand side of the image. Exposition time was 1 minute for all the membranes. Molecular weight ladders are as follows: (L1) Supersignal molecular weight protein ladder (150 kDa, 100 kDa, 80 kDa, 60 kDa, 50 kDa, 40 kDa, 30 kDa, 20 kDa), and (L2) multicolor protein ladder (260 kDa, 135 kDa, 95 kDa, 72 kDa, 52 kDa, 42 kDa, 34 kDa, 26 kDa, 17 kDa, 10 kDa). Expected protein molecular weights are: 98 kDa for FURIN (expected band indicated with an arrowhead), 41 kDa for NFE2, and 36 kDa for GAPDH.
